# Progress Evaluation for the Restaurant Industry Assessed by a Voluntary Marketing-Mix and Choice-Architecture Framework That Offers Strategies to Nudge American Customers toward Healthy Food Environments, 2006–2017

**DOI:** 10.3390/ijerph14070760

**Published:** 2017-07-12

**Authors:** Vivica Kraak, Tessa Englund, Sarah Misyak, Elena Serrano

**Affiliations:** 1 Department of Human Nutrition, Foods, and Exercise, Virginia Tech, Blacksburg, VA 24061, USA; retessa@vt.edu (T.E.); serrano@vt.edu (E.S.); 2 Virginia Cooperative Extension’s Family Nutrition Program, Department of Human Nutrition, Foods, and Exercise, Virginia Tech, Blacksburg, VA 24061, USA; smisyak@vt.edu

**Keywords:** restaurants, choice-architecture, healthy food environments

## Abstract

Consumption of restaurant food and beverage products high in fat, sugar and sodium contribute to obesity and non-communicable diseases. We evaluated restaurant-sector progress to promote healthy food environments for Americans. We conducted a desk review of seven electronic databases (January 2006–January 2017) to examine restaurant strategies used to promote healthful options in the United States (U.S.). Evidence selection (*n* = 84) was guided by the LEAD principles (i.e., locate, evaluate, and assemble evidence to inform decisions) and verified by data and investigator triangulation. A marketing-mix and choice-architecture framework was used to examine eight voluntary strategies (i.e., place, profile, portion, pricing, promotion, healthy default picks, priming or prompting and proximity) to evaluate progress (i.e., no, limited, some or extensive) toward 12 performance metrics based on available published evidence. The U.S. restaurant sector has made limited progress to use pricing, profile (reformulation), healthy default picks (choices), promotion (responsible marketing) and priming and prompting (information and labeling); and some progress to reduce portions. No evidence was available to assess progress for place (ambience) and proximity (positioning) to promote healthy choices during the 10-year review period. Chain and non-chain restaurants can apply comprehensive marketing-mix and nudge strategies to promote healthy food environments for customers.

## 1. Introduction

In 2014, more than two thirds (70.7 percent) of adults [1] and one third (32.4 percent) of children and adolescents, ages 2–19 years, were overweight or obese [2]. The frequent consumption of restaurant products by children, adolescents and their parents in the United States (U.S.) is a significant driver of poor diet quality, obesity and diet-related non-communicable diseases (NCDs) [3,4]. Nearly two-thirds of American adults visit quick-service restaurants (QSR) and 40 percent visit fast-casual restaurants (FCR) every week [5], and 30–40 percent of children and adolescents visit QSR every day [6]. 

Trend data suggest that most food and beverage products from U.S. chain and non-chain restaurants exceed the Dietary Guidelines for Americans (DGA) [7] recommendations for total calories, fat, sugar and sodium. American children and adolescents consume 10–30 percent of their dietary sodium [8], 20–35 percent of fats and added sugars [9,10], and 13 percent of total daily calories from burgers, pizza, fries, dairy desserts, and sugar-sweetened beverages (SSBs) purchased at restaurants [6,7,8,9,10]. For example, young Americans who purchase an SSB with a QSR bundled meal consume about 179 more calories [11] and are more likely to exceed the DGA recommendation of ≤25 grams (≤6 teaspoons) of added sugars/person/day [7].

Some data show that American children and adolescents, ages 4–19 years, reduced their fat, sugar and sodium consumption at QSR chains between 2003 and 2010 [12]. Nevertheless, the average daily sodium intake of Americans, ages 2 and older, was 3409 milligrams (mg) in 2013–2014, which exceed the Healthy People 2020 recommended target of 2300 mg/person/day, and the source of foods with the highest sodium density (mg sodium/1000 calories) consumed by children were full-service restaurants [13]. Efforts aimed at reducing the frequency and amount of purchasing and consuming food, beverage and meal products high in fat, sugar and sodium (HFSS) at chain and non-chain restaurants may be facilitated through behavioral economics strategies to help reduce obesity and NCD rates [14]. 

Choice architecture is an approach used to design choices in various environments to influence people’s decisions and behaviors [15]. Nudge or nudging are used to describe different forms of choice architecture. Nudging is defined by Thaler and Sunstein [16] as “*Any aspect of choice architecture that alters people′s behavior in predictable ways without restricting any options or significantly changing their economic incentives*” such as time or money. Nudge theory, based on decades of psychology and behavioral economics research, advances the concept of libertarian paternalism that favors using cognitive biases and “rules of thumb” to facilitate people′s decision-making in the marketplace. However, nudge actions alone are limited because they exclude pricing strategies that are recognized as a powerful intervention to reduce obesity risk and health disparities among low-income populations [17,18].

The U.S. restaurant sector could encourage healthy choices by combining marketing-mix strategies (i.e., product, place, price and promotion) [19,20] and choice-architecture strategies (i.e., place, healthy default picks, promotion, priming or prompting and proximity) [21,22]. A companion paper describes a three-step process whereby we developed a novel marketing-mix and choice-architecture framework for the U.S. restaurant sector to promote healthy food environments. Step one involved conducting a systematic evidence review of various choice-architecture typologies and taxonomies used to categorize strategies that cue healthy behaviors in microenvironments [23]. Of the five typologies identified, three were adapted and combined with marketing-mix principles to highlight eight strategies (i.e., place, profile, portion, pricing, promotion, healthy default picks, prompting or priming and proximity) [23]. 

This “proof of concept” framework had some overlap between certain strategies (e.g., place) that appeared in both the marketing mix and choice architecture with regard to changing the internal or external features (e.g., atmosphere or ambience) of a setting or place [23]. The need to combine the strategies was evident due to the limitations of nudge strategies that explicitly exclude pricing manipulations, which are a classic feature of the conventional commercial marketing mix. Pricing strategies are a powerful intervention to reduce socioeconomic inequities associated with obesity and diet-related NCDs [24]. Step two involved conducting a comprehensive evidence review between January 2006 and December 2016 to identify U.S. recommendations for the restaurant sector organized by strategy. Step three entailed developing 12 performance metrics for the eight strategies to promote healthy food environments. No evaluation has yet examined how the U.S. restaurant sector has combined and used these marketing-mix and choice-architecture strategies to promote healthy food environments for Americans in relation to recommendations issued by industry, government and public health authoritative bodies [23].

### Study Purpose

The question that guided this research was: *What progress was made by U.S. chain and non-chain restaurants and the restaurant sector to create healthy food environments for American children, adolescents and parents between 2006 and early 2017?* Healthy food environments are defined as the economic, policy and sociocultural conditions, sectors and settings that offer people access to healthy and affordable foods and beverages to prevent or help reduce obesity and diet-related NCDs [25]. The restaurant industry′s use of marketing-mix and choice-architecture or nudge strategies are important components to create and promote a healthy food environment. We use the results to suggest future policies and actions that individual restaurants and the U.S. restaurant sector could take to promote healthy food environments for Americans. 

## 2. Materials and Methods

### 2.1. Literature Search Strategy and Evidence Review

Table 1 summarizes eight strategies (i.e., place, profile, portion, pricing, promotion, healthy default picks, priming or prompting and proximity) and 12 performance metrics used to evaluate the U.S. restaurant sector’s collective actions. We accessed eight electronic databases for published peer-reviewed studies; gray-literature reports and websites (e.g., industry, government, non-governmental organization and foundations); and media stories. Table 2 summarizes the search strategy based on the LEAD principles (i.e., *locate*, *evaluate* and *assemble* evidence to inform *decisions*), including the methods used to acquire, select, categorize and analyze the evidence [26]. The review was conducted starting 1 January 2006 through 30 January 2017. The evidence sources (*n* = 84) are summarized in three supplemental evidence tables: peer-reviewed articles (*n* = 49); gray-literature reports (*n* = 18); and relevant media stories (*n* = 27) used to evaluate U.S. restaurant progress for each strategy in the framework. Table 3 provides definitions for and examples of different types of U.S. chain and non-chain restaurants. We used the National Restaurant Association (NRA) [27] and Technomic, Inc. [5] definitions for various restaurant sub-categories including: limited-service restaurants (LSR), which represent QSR and FCR; and full-service restaurants (FSR). Table 4 summarizes the recommendations for the U.S. restaurant sector issued by 16 authoritative government, industry and public health bodies [23]. 

### 2.2. Evidence Analysis

The evidence acquired was analyzed by the investigators between 1 December 2016 and 15 March 2017 using five qualitative research criteria [28] and verified by data and investigator triangulation. The criteria used to evaluate progress toward each of 12 performance metrics developed from a review of 16 authoritative reports [23] listed in Table 4. The performance metrics summarized in Table 1 were based on the following criteria: *none* (no evidence of actions), *limited* (evidence of 1–2 specific actions), *some* (evidence of 3–4 specific actions) and *extensive* (evidence of 5 or more actions). Given the qualitative nature of the evidence reviewed, we reviewed progress based on the range and median actions taken by individual restaurants and the restaurant sector for each marketing-mix and nudge strategy. 

## 3. Results

Summarized below and in Table 5 are the results for whether and how restaurants applied each marketing-mix or nudge strategy to promote healthy food environments between 2006 and early 2017. Supplemental Table S1 summarizes the peer-reviewed articles (*n* = 49); Table S2 summarizes the gray-literature reports (*n* = 18); and Table S3 summarizes the relevant media stories (*n* = 27) used to evaluate U.S. restaurant progress for each strategy in the marketing-mix and choice-architecture framework during the designated time frame.

### 3.1. Strategy 1: Place—No Evidence to Assess Progress

Place involves restaurants changing the ambience or atmosphere (e.g., music or lighting) of the premises to make healthy choices more visually or aesthetically appealing to customers. No evidence was identified to assess progress made by QSR, FCR or FSR chains to use place to promote healthy choices for customers during the review period. 

### 3.2. Strategy 2: Profile—Limited Progress

Profile represents changes to improve the nutritional composition, smell, taste, texture and flavor of food and beverage products that meet recommended nutrient targets. Limited progress was made by the restaurant sector based on a review of 32 studies (*n* = 25 peer-reviewed articles) and (*n* = 7 gray-literature reports) that examined U.S. restaurants’ reformulation, with a special emphasis on improving healthy offerings for children and adolescents. The studies described below examined changes in two domains: (1) changes in nutrient profiles of products for total calories, fat, saturated fat, sugar and sodium; and (2) changes in menu quality to meet recommended healthy criteria or guidelines during the review period. 

#### 3.2.1. Nutrient-Profile Changes to Restaurant Offerings

Eighteen of 25 studies [29,30,31,32,33,34,35,36,37,38,39,40,41,42,43,44,45,46] found that QSR, FCR and FSR chains made either a modest or no reduction in total calories, fat and saturated fat or products between 2006 and 2015. In 2006, Serrano and Jedda [29] found that children’s meals sold at non-chain restaurants offered a higher percentage of calories from total fat and saturated fat compared to QSR meals. In 2007, Dumanovsky et al. [30] documented that New York City (NYC) customers purchased about 827 calories/meal, and some QSR provided more than 930 calories/meal, well above the recommended ≤700 calories/meal [24] (Table 1). Bruemmer et al. [31] found that between 2009 and 2010, LSR and FSR chains had lowered the energy content of meals by about 19 calories/item and 73 calories/item, respectively, though restaurants still exceeded the calorie and saturated fat targets half of the time [31]. 

Between 2006 and 2010, Bauer et al. [32] found no change in the calorie content of entrées and beverages, a decrease in calories for side-item dishes, and an increase in calories for desserts. In 2010, Urban et al. [33] found that the QSR, FCR and FSR chain offerings across three states contained at least 100 calories/portion more than the stated energy content [34]. In 2010, Wu and Sturm [35] found that restaurants not offering voluntary menu labeling or nutrition information for children′s meals had higher fat and saturated fat content. Wu and Sturm [36] found no significant change in the energy content of children′s meals one year after enactment of the 2010 national restaurant menu-labeling law.

In 2013, Cardello [47] uniquely found that 17 of 21 QSR, FCR and FSR chains had expanded the availability and sales of lower-calorie items between 2006 and 2011 that were linked to increased customer purchases. Urban et al. [37] documented that small and large non-chain ethnic restaurants (e.g., Mexican and Chinese) sold an average of 1327 calories/meal, and three-quarters of these restaurants sold at least 50 percent of the recommended energy requirement for an average adult (2000 calories/day) in one meal. Auchincloss et al. [38] observed an average energy content of 800 calories/entree at QSR, FCRs and FSR that met healthy criteria 47 percent of the time; 30 percent of the *à la carte* entrees exceeded the daily recommended level for saturated fat; and only 20 percent of items met the dietary fiber target. Bleich et al. [39] found no meaningful calorie reduction for existing restaurant menu items offered by 66 restaurant chains between 2012 and 2013, but a modest 26–67 calorie decline for newly introduced items. In a follow-up study of 37 QSR and FCR chains, Jarlenski et al. [40] documented an average 22-calorie reduction in menu items between 2012 and 2014. 

Between 2006 and 2013, Urban et al. [41] documented a reduction in trans fats for selected items but no reduction in saturated fat for restaurant offerings. Between 2011 and 2014, Urban et al. [42] found that non-chain restaurants offered an average of more than 1200 calories, and 92 percent of non-chain restaurants exceeded the recommended calories/meal for adult women., Schoffman et al. [43] documented that in 2014 FCR provided more high-calorie entrees (≥750 calories/item) compared to QSR (≤600 calories/item). Sliwa et al. [44] found that a majority of children’s meal combinations in 2014 met the recommended ≤600 calories/meal, but fewer than a third (31.9 percent) met the recommendation for items ≤35 percent of calories from fat and ≤10 percent of calories from saturated fat. Bleich et al. [45] observed a 30-calorie decline for newly introduced items, but most items sold between 2012 and 2015 had no significant calorie reduction. 

Moran et al. [46] found that 45 chain restaurants (*n* = 23 QSR, 19 FCR and six FSR) had increased beverage calories offered with children′s menu items (*n* = 4016) between 2012 and 2014. From 2012 to 2015, these chains made no significant changes in beverage calories, total calories or saturated fat for children‘s meals and other menu items. Restaurants that participated in the NRA’s Kids LiveWell Program (*n* = 15) were more likely to have reduced children‘s entrées by about 40 calories/meal between 2012 and 2014 [46]. 

Nine studies [36,38,41,44,48,49,50,51,52] found either no reduction, a modest decline, or an increase in the sodium content of menu offerings for children, adolescents and parents between 2006 and 2014. Rudelt et al. [48] found no change in sodium of offerings by 2010 among eight QSR chains. Jacobson et al. [49] documented a 2.6 percent increase in sodium in 2011 among 16 QSR and FCR chains. Auchincloss et al. [38] documented that 30 percent of entrees exceeded the daily sodium recommendation in 2011 among 22 QSR, FCR and FSR chains. Wu and Sturm [36] found no significant change in the sodium of meals sold in 2011at 213 unidentified restaurants. Urban et al. [41] found no sodium reduction for items between 2006 and 2013 in three major LSR chains. Ahuja et al. [50] found that 88 percent of items exceeded the FDA’s sodium limit in 2013 at four QSR chains and non-chain Chinese restaurants. 

Gray-literature reports concurred with the findings summarized above. The Center for Science in the Public Interest (CSPI) [51] conducted an evaluation in 2013 that found a 2.6 percent decrease in the average sodium content across all meals, but 44 percent of children’s meals contained more than 1200 mg sodium among 17 QSR, FCR and FSR chains. Between 2012 and 2014, the CSPI [52] documented a one percent sodium decline across all menu items, but children’s meals and side dishes revealed a 2.8 percent increase in sodium at 25 QSR, FCR and FSR chains. In 2014, a separate analysis conducted by Sliwa et al. [44] documented that less than one third (31.9 percent) of children’s meal combinations provided 770 mg or less of sodium at 20 QSR and FSR chains.

#### 3.2.2. Menu-Quality Changes to Restaurant Offerings

Nine studies [53,54,55,56,57,58,59,60,61] revealed that between one and 13 percent of meals sold to children, adolescent and their parents met healthy criteria recommended by authoritative bodies. Kirkpatrick et al. [53] (*n* = 5 QSR chains) found that in 2006 children’s menu items scored higher than adult items, and those labeled “healthy or nutritious” had a higher HEI-2005 score than the full-menu items. Nevertheless, all items had poor nutritional scores despite variable diet quality [53]. O′Donnell et al. [54] (*n* = 10 QSR chains) documented that in 2007, only three percent of children’s meal combinations met the USDA’s nutrition standards. In 2008, Wootan et al. [55] found that six of 25 restaurant chains did not offer any children’s meals, and 93 percent of 19 different children’s restaurants meals provided excessive calories, saturated fat and sodium. 

Harris et al. [56] (*n* = 12 QSR chains) documented that in 2009 less than one percent (12 out of 3039 children’s meals) met recommended nutrient targets using the nutrient profiling index. Hearst et al. [57] observed an increase in the HEI-2010 score for six of eight QSR chains between 2009 and 2010, yet the overall nutrition quality remained poor for most menu offerings. Krukowski et al. [58] (*n* = 130 local QSR chains and non-chains) found that only 13 percent of children′s menus provided healthy options between 2009 and 2010. Between 2012 and 2013, three studies [59,60,61] revealed that between one and 11 percent of children’s meals met recommended nutrition criteria. 

### 3.3. Strategy 3: Reduce and Standardize Portions—Some Progress

Portion changes involve restaurants reducing or standardizing meal size to meet healthy dietary guidelines and influencing customers′ expectations about an appropriate single serving. Four studies suggested some progress was made to reduce meal portions to improve the nutrient profile of children’s meals [62,63,64,65]. The first study suggested that reducing the portion size of fries in a children’s meal bundle could lead to 98 fewer calories consumed [62]. Restaurant owners identified several factors that affected the reduction of children’s meal portions including: potential revenue losses, logistical barriers, lack of customer demand, different portion sizes, and desire to be a responsible industry leader [63]. However, a rural [64] and urban [65] study demonstrated that portions could be reduced if technical assistance and public recognition were offered to promote healthy restaurant programs. The fourth study was conducted in an urban city of Texas, and 12 of 17 restaurants reported using the healthy children’s menus two years after the intervention had ended [66]. 

### 3.4. Strategy 4: Pricing—Limited Progress 

Pricing involves restaurants influencing point-of-purchase behaviors to encourage customers to select healthy products and smaller portions while retaining profitable sales and revenue. Four studies suggested that increasing the price for healthier children’s meals at two FSR chains could encourage parents to purchase the healthy meal options [67,68,69,70]. This marketing-mix strategy received limited progress because no major QSR, FCR or FSR chains had reduced price promotions on large portions and package sizes or had used competitive pricing to encourage healthy meals during the review period. 

A 2009 study compared price differences between children’s meals rated to be healthy versus unhealthy in 15 FSR chains in Arkansas but found no significant price difference [67]. In 2012, Silver Diner, a New England FSR chain, made healthy children’s menu improvements [68]. Despite the children′s meal prices increasing by about 80 cents for breakfast and 20 cents for non-breakfast meals, the restaurant’s revenue increased for parents ordering healthy children′s meals and healthy-default side dishes. While the healthy children′s menu led to a reduction in calories ordered from 684 to 621 calories/meal [68], and changes were sustained over two years [69], total energy still exceeded the NRA’s and expert panel recommended target of ≤600 calories/meal. A third study examined The Walt Disney Company’s restaurant sales (2010–2012) and found that customer purchases were not influenced by increasing the cost of more nutritious children’s meals [70].

### 3.5. Strategy 5: Promotion–Limited Progress 

Promotion is a strategy that involves restaurants adhering to responsible marketing practices that promote products that meet recommended nutritional guidelines while concurrently restricting the promotion of HFSS products [23] (Table 1). For evidence published from January 2006 through January 2017, eight peer-reviewed studies (Table S1) and four gray-literature reports (Table S2) showed limited U.S. restaurant sector progress to implement comprehensive marketing practices to promote healthy meals and restrict HFSS product marketing. The evidence in the section below summarizes the U.S. restaurant sector′s use of television (TV) advertising, toy premiums and incentives, brand mascots and media characters, celebrity endorsement, and child-directed marketing on the exterior of restaurants. 

#### 3.5.1. Responsible Marketing of Healthy Meals

In 2006, the NRA joined with Healthy Dining to offer a website to help parents select healthy children’s meals [71]. In 2010, the NRA companies were encouraged by First Lady Michelle Obama to market healthy options to children [72]. The NRA responded by establishing the Kids LiveWell Program in 2011 [73] to encourage participating restaurants to offer at least one healthy children’s meal that provided ≤600 calories and aligned with recommended fat, saturated fat and sodium targets [74]. The Kids LiveWell Program also encouraged restaurants to offer more fruits and vegetables, whole grains, lean protein and low-fat dairy [74]. The NRA’s program was honored by the industry in 2013 [75]. However, an analysis of nearly 3500 children’s meals at 34 restaurants in 2013 found that only three percent met all of nutrition all of the program’s guidelines [59]. A separate analysis of more than 4000 children’s menu items found that participating restaurants had not significantly reduced calories, saturated fat and sodium in children’s entrees, beverages, side dishes and desserts between 2012 and 2015 [46]. Moreover, Kids LiveWell restaurants had replaced soda in children′s meals with other sugary beverages (e.g., flavored milk and sports drinks) [46]. 

In 2011, Jack in the Box announced that it would no longer offer toys with children’s meals [76], followed by Yum Brands Announcement that Taco Bell would discontinued providing toys with children’s meals at 6000 restaurants in 2013 [77]. These actions set important precedents since toy giveaways represent $360 million spent by the restaurant industry on child-directed marketing [78]. 

In 2012, McDonald’s started a “balanced eating and active play” campaign to promote healthier Happy Meals [79,80] and announced five commitments in 2013 to improve the nutritional quality of the meals that was brokered through an agreement facilitated the Alliance for a Healthier Generation and Clinton Global Initiative [81]. By 2014, McDonald’s and Burger King had adopted the CFBAI′s uniform nutrition criteria for products marketed to children under 12 years [82,83]. A 2015 progress evaluation [84] showed that McDonald′s had made progress between 2013 and 2014 to implement its commitments toward the 2020 goals [85]. In 2015, Walt Disney started using a “Mickey Check” icon to promote healthy options through packaging that aligned with its healthy restaurant guidelines [86]. 

#### 3.5.2. Restrict Marketing of HFSS Products

In 2009, the QSR sector spent more than $4.2 billion that increased to $4.6 billion in 2012 to advertise through TV, digital, mobile and social media [78]. A 2012 FTC report that analyzed QSR expenditures in 2006 and 2009 found that these companies collectively spent $714 million on youth marketing in 2009, a decrease from $733 million in 2006 [78]. Between 2006 and 2009, the FTC reported a drop in child-directed QSR expenditures, attributed to reductions in toy premium spending, despite an increase in teen-directed QSR expenditures due to higher TV expenditures, and a modest increase in radio and new media advertising [78,87]. Readers should note that these reported marketing expenditures from 2009 through 2012 are not comparative because they are not adjusted for inflation. 

From 2005 through 2012, TV advertising by restaurants represented a high proportion of HFSS food and beverage products viewed by 21 percent of preschoolers, 34 percent of children ages 2–11 years, and 39 percent of adolescents ages 12–17 years [87]. Between 2014 and 2015, young people were exposed to more QSR TV advertisements than any other food category, representing 29 percent of TV advertisements viewed by children and more than 33 percent viewed by adolescents [88]. 

In 2009, the expenditures on celebrity marketing by leading QSR chains was $1 million dollars to children and $7 million dollars to adolescents [78]. After 2009, McDonald’s but not Burger King had changed the company’s communications messaging to promote apple dippers and low-fat milk in children’s meals [89]. Between 2010 and 2013, African-American and Latino children and adolescents were still targeted by McDonald′s TV advertisements, compared to white children and adolescents [56,90], despite earlier recommendations to stop targeted marketing [24] (Table 3). Moreover, more than 20 percent of 6716 QSR chains located near public middle and high schools continued child-directed marketing in 2013 either inside or on the exterior of the chains targeting African-Americans, rural and middle-income communities [91]. 

In 2010, Santa Clara County and San Francisco passed an ordinance that required restaurants to distribute toys or incentives to children in restaurant meals only if they met nutrition standards [92]. An evaluation documented that McDonald′s and Burger King had not expanded the number of healthy meal options or improve the nutritional quality of meals to meet these recommended standards [93]. These QSR chains responded to the ordinance by selling the toys separately from meals, and neither chain changed their menus to meet the specified nutrition criteria [94]. Offering a toy with smaller menu portions could be a potential strategy to provide a healthy meal and toy incentive [95]. However, a separate study identified McDonald’s as the leading restaurant that used toy premiums to market to young children between 2013 and 2014, and reported that children’s knowledge of fast food toys was associated with eating more frequently with parents at McDonald’s [96]. 

In 2011, several restaurants continued to use marketing practices that were not covered by the CFBAI’s voluntary pledges including toy premiums, licensed media characters (e.g., *Dora*, *Scooby Doo* and *Shrek*) and brand mascots (e.g., *Ronald McDonald* and *Happy*, and *Chuck E Cheese*) [97,98]. McDonald’s continued to advertise to children that emphasized toy premiums rather than food [99]. 

In 2010 San Francisco and Santa Clara County ordinances [100] passed ordinances that required restaurants to meet specific nutrient criteria for children’s meals that offered toy premiums. In 2015, the NYC Council proposed the “Healthy Happy Meals” bill to improve the nutritional quality of children’s combo meals that would require specific nutritional criteria if toy premiums were offered. The CFBAI admonished McDonald’s in 2015 to shift its TV advertising targeting children to food instead of the toy [101]. But in 2016, the NYC Health Commissioner rejected the Healthy Happy Meals bill, which McDonald′s spent $528,000 dollars to lobby members of the NYC Council to oppose it [102], on grounds that restaurants would not comply with the ordinance, and it could not be easily enforced [102]. 

### 3.6. Strategy 6: Establish Healthy Default Picks—Limited Progress 

Healthy default picks involve establishing healthy default choices that are attractive and convenient for customers. Three studies supported limited progress made by selected restaurants to expand default healthy side dishes (e.g., fruits and vegetables) and beverages (e.g., low-fat or non-fat milk, 100 percent juice and water) with children’s meals [70,103,104]. The first study surveyed children and adolescents (*n* = 1178; ages 8 to 18 years) and found they would select fruit or vegetables to replace fries, representing a 170-calorie reduction [103]. However, of 20 restaurants surveyed, 60 percent of QSR and 70 percent of FSR chains did not serve fruit or vegetable defaults with children′s meals [103]. The second study examined sales data for children’s meals sold by Walt Disney between 2010 and 2012 [70], and found that 48 percent and 66 percent of customers accepted the healthy-default side dishes and beverages, respectively, for children’s meals that reduced calories (21.4%), fat (43.9%) and sodium (43.4%) purchased but not added sugars [70]. The third study surveyed mothers of children, ages 3–8 years, and found that healthy default choices for bundled meals were favored if mothers could choose the items [104].

Gray-literature reports and media stories reported mixed results for restaurants establishing healthy defaults during the review period. In 2013, CSPI released a restaurant monitoring report of 50 restaurant chains that found only Subway offered low-fat milk or bottled water as default beverages and apple slices as the default side dish [59]. In 2014, CSPI initiated a social media advocacy campaign to encourage all restaurant chains to remove soda and other SSBs from children’s meals [105]. In 2015, McDonald’s announced five commitments to offer only water, milk and juice as the Happy Meals’ beverage; and a side salad, fruit or vegetables to replace fries in value meals [106]. Between 2015 and 2016, 10 chain restaurants (i.e., Applebee′s, Burger King, Chipotle, Dairy Queen, IHOP, Jack in the Box, McDonald’s, Panera Bread, Subway and Wendy’s) announced plans to remove soda as the default beverage for children’s meals [107,108]. Between 2015 and 2016, the City Councils in Davis and Stockton (California) enacted ordinances to establish milk and water as default beverages for children’s meals [109,110]. By the end of 2016, most U.S. chain restaurants and independently owned non-chain restaurants continued to offer SSBs as the default beverage for children’s meals [111]. 

The food service industry announced a goal in 2009 to double the use of fruit and vegetables in menus and catering by 2019 [112]. Between 2013 and 2016, only a few QSR chains (i.e., McDonald’s [113] and Subway [114], and the FCR chain, Panera Bread [115]), had expanded fruits and vegetables as healthy side dishes for children′s meals. The National Fruit and Vegetable Alliance released a scorecard in 2010 [116] and 2015 [117] for the restaurant sector and other food service establishments. In 2010, the restaurant sector received D and C grades for limited provision of fruits and vegetables, respectively; and a B- in 2015 for improved yet limited actions to expand the availability and sales of fruits and vegetables in entrees, value meals, and as side dishes for children’s meals. 

### 3.7. Strategy 7: Use Priming or Prompting for Healthy Choices—Limited Progress

Priming or prompting encourages customers to purchase and consume healthy options or discourages less healthy options at restaurants. These strategies may include restaurants providing numerical or interpretive information through calorie labelling on menus, menu boards or online; front-of-packaging symbols or logos; health or safety warning messages or symbols on products to influence customers to avoid nutrients of concern such as excessive dietary sodium or added sugars; and restaurant staff offering customers verbal cues about healthy options. 

In 2010, the Patient Protection and Affordable Care Act included a mandatory national restaurant menu-labeling provision [118] that the NRA supported [119]. The law required U.S. restaurants with 20 or more outlets to modify their menu offerings and marketing practices that the FDA will enforce starting May 2018 [120]. The law also required restaurants to provide calorie labeling, specific language for the daily calorie requirements of adults and children, and additional nutrition information upon request [121,122]. 

Four studies (Table S1) summarize results for restaurant chains that provided calorie menu labeling or nutrition information to customers during the review period., Bruemmer et al. [31] found that between 2009 and 2010 restaurant menu labeling in Washington state may have influenced 37 LSR and FSR chains to lower the energy content of meals by about 19 calories/item and 73 calories/item, respectively. Nevertheless, these restaurants still exceeded the calorie and saturated fat targets half of the time [31]. Wu and Sturm [35] examined 400 restaurant chains between February and May 2010, and found that less than two-thirds offered customers nutrition information. 

Moreover, restaurants that did not voluntarily provide menu labeling or nutrition information sold children’s meals that were higher in fat and saturated fat [35]. In a follow up study, Wu and Sturm [36] found no change in the energy or sodium content of children’s meals at 213 restaurant chains one year after the 2010 national menu-labeling law enactment. Bleich et al. [123] examined outlets from 66 restaurant chains between 2012 and 2014, and found that the mean per-item calorie content of food items sold by restaurants that voluntarily implemented menu labeling before it was mandated offered lower-calorie items compared to non-participating restaurants.

Warning labels are a type of priming or prompting used to discourage customers from purchasing or consuming products that are high in sodium or sugar. In December 2015, the NYC Department of Health and Mental Hygiene enacted an ordinance that required restaurant chains with at least 15 U.S. locations to post a black and white salt icon to warn customers about menu items providing 2300 milligrams of sodium [124,125]. The NRA sued the NYC legislature over this sodium-labeling mandate [126], although Applebee’s, TGI Friday’s and Subway voluntarily complied with the NYC ordinance [127]. In May 2016, a court ruled in favor of the NYC ordinance to use the high-sodium warning label [127]. 

### 3.8. Strategy 8: Proximity—No Evidence to Assess Progress

Proximity involves influencing the food and beverage choices and behaviors of restaurant customers by placing healthy choices at eye level and closer to point-of-purchase, and placing less healthy choices further from reach and point-of-choice or point-or-purchase. No evidence was identified to assess progress for restaurant chains using proximity or positioning to promote healthy choices for customers during the review period. 

## 4. Discussion

This evaluation found that the U.S. restaurant sector made *limited progress* to use pricing, profile (reformulation), priming (healthy defaults), promotion (responsible marketing) and prompting (labeling), and *some progress* to reduce portions to encourage healthy choices for American consumers during the 10-year period reviewed (2006 to early 2017). No evidence was available to assess progress for place (ambience and atmospherics) and proximity (positioning). Table 5 and the section below summarize suggested actions that the U.S. restaurant sector and individual companies could take to use comprehensive voluntary marketing mix and nudge interventions to promote healthy food environments for Americans. 

To improve the place, restaurants can create an ambience or atmosphere to support a healthy eating culture, and use lighting and visual cues to make healthy choices appealing to young people and parents. To improve the profile, the NRA and CFBAI could encourage restaurants to offer 100 percent of entrees, bundled children′s meals, value meals, side dishes, beverages and desserts that meet nutrition targets recommended by expert bodies [23]. This analysis found that 18 out of 25 studies documented either a modest or no reduction in total calories, fat and saturated fat in products served and sold. Nine studies found either no reduction, a modest decline, or an increase in the sodium content of menu offerings. Results from three studies revealed that less than 13 percent of children’s meals met healthy criteria or guidelines recommended by authoritative bodies. 

An important insight about the nutritional profiles of restaurant products is that investigators have used a variety of approaches to determine whether restaurants have made meaningful changes that meet recommended nutrient targets. Table 2 and Supplemental Table S1 describe diverse data sources, many assessment tools and different dietary standards used to conduct monitoring and evaluations. Recent studies of nutrient composition changes have used the Menustat database, which is the most comprehensive public nutrition database for U.S. restaurant food and beverage products. However, there are still information gaps for serving sizes and weights for this database [128]. Restaurants could provide the NYC Department of Health with updated information on a periodic basis to enable researchers to conduct independent evaluations of the nutrient profiles of restaurant products [128]. 

Recent research on trends in calorie changes made by large U.S. chain restaurants (2008–2015) [129] suggests that restaurants are responding to the enforcement of the restaurant calorie labeling law to start in May 2018 [120]. However, restaurants will not be required to disclose other nutrients of concern including sodium, saturated fat and added sugars. This analysis showed that many large chain restaurants have not made meaningful progress to reduce the fat, saturated fat, added sugars and especially the sodium content of offerings to align with recommended targets for meals sold to children, adolescents and parents. Technical assistance is available to help restaurants meet these nutrient targets [130]. Some research suggests that consumers will accept restaurants making modest and gradual recipe changes to items over time to reduce total calorie, fat and sodium intake [131].

Restaurants could also reduce and standardize *portions* to meet recommended nutrient targets for calories, fat, saturated fat, added sugars and sodium. The limited evidence from this evaluation suggested that the FSR chains, Silver Diner and Walt Disney, had made positive changes. However, the children’s meals still exceeded the recommended ≤600 calories/meal target and did not fully meet other nutrient targets [68,69,70]. Moreover, most parents are unaware of the energy recommendation of ≤600 calories/children’s meal [132], and many restaurant offerings exceed the recommended 700 calories/meal target for teens and adults. 

Restaurants could make menu changes by adding new healthy items and changing existing items to have a broader impact to improve the dietary quality of children’s restaurant meals [133]. Through the Kids LiveWell Program, the NRA should show leadership to incentivize members to reduce and standardize portions for *à la carte* components of children′s “bundled meals” to meet 100 percent of their own nutrient criteria. An expert panel recently proposed the following standardized calorie portions: 300 calories for main dishes; 100 calories for fried potatoes; 150 calories for soups, appetizers and snacks; and 150 calories for vegetables and salads with added ingredients or sauces [134].

The results from three pricing studies in selected restaurant settings suggest that parents will pay more for smaller portions of healthy meals and that restaurant businesses can profit from the increased purchases of healthy meals. Future research is needed across QSR, FCR and FSR chains, and non-chain restaurants, to understand how restaurant owners can use proportionate pricing, and combine pricing changes with other nudge and marketing strategies, to increase healthy product sales [135,136]. 

To improve promotion, restaurants should adopt comprehensive responsible food and beverage marketing policies that promote healthy offerings while also restricting HFSS food and beverage marketing through integrated marketing communications. This is an important finding because many QSR still market HFSS food and beverage products to preschool-aged children [89], young children are more likely to recall meals with toy premiums [137], and there is a direct relationship between TV advertising and fast food consumption by children under 5 years [138]. Additionally, more than a third of young people are exposed to more QSR TV advertisements than any other food category [87,88]. 

Our analysis found that between 2006 and early 2017, neither the CFBAI, Kids LiveWell Program nor any major chain restaurant had pledged to extend healthy nutrition criteria to food marketing practices targeted to adolescents, ages 12–18 years. A 2017 Rudd Center survey of 3600 parents with children and adolescents, ages 2 to 17 years, revealed that parents believe that young people′s easy access to QSR foods and beverages contributed to undermining their eating behaviors [139]. Three quarters (74 percent) of parents surveyed want restaurants to enforce healthy children′s meal policies [139]. Future evaluations are needed to explore how restaurants can combine responsible marketing in the form of many promotional strategies (e.g., toy incentives, signage and colorful placemats) with other marketing-mix and nudge strategies (e.g., pricing and prompting) to encourage children and their parents to purchase healthy meals that meet recommended calorie and other nutrient targets [140]. 

To encourage healthy default picks, restaurants should establish policies that offer fruit or vegetable options as the default side dishes, low-fat milk or water as default beverages, and discontinue policies that promote free unlimited SSB refills. Healthy default beverages are expanding in some locations, such as Santa Clara County (California) that passed a law in 2017 that required restaurants to provide healthy default beverages with children’s meals [141]. Nevertheless, our review showed that U.S. chain and non-restaurants have made limited progress to voluntarily establish healthy default picks, such as the replacement of fries with fruits or vegetables as a side dish; and no major chain has announced a policy to offer free unlimited self-serve water refills instead of full-calorie SSB refills. American customers who select unlimited SSB refills in chain restaurants may consume up to 250 beverage calories more than customers who do not choose refills [142]. Adopting both policies are important voluntary efforts that restaurants can make to change social norms about health default choices. 

To maximize the use of priming and prompting, restaurants could partner with researchers to determine the effectiveness of training and incentivizing paid staff to prompt customers to select the nutrient-dense, healthy-default options. The need for such research is important because our study found that a majority of chain and on-chain restaurant meals sold to children, adolescents and their adult parents do not meet recommended nutrient targets for fat, saturated fat, added sugars and sodium. Moreover, consumer studies have shown either a positive purchasing trend [143] or no association between U.S. restaurants providing nutrition information or different menu labeling formats to encourage customers to reduce their calories purchased or consumed [144,145,146,147]. 

In March 2017, Panera was the first FCR chain to voluntarily disclose the added sugars and calorie content of sodas and other self-serve fountain beverages to help customers make informed choices [148]. In May 2017, the New York City Department of Health and Mental Hygiene reported that nine out of ten chain restaurants had complied with the sodium warning rule requiring warning labels to be posted where customers placed their orders as a prompt to remind them that a high sodium intake can increase their blood pressure and risk of a heart attack [149]. Given these recent policy changes that occurred after our designated review period, we were unable to identify evidence to suggest that calorie labeling for soda and fountain beverages or the sodium-warning label enacted in NYC had inspired restaurants to reformulate high-sodium products or represented an effective priming or prompting influence on customers’ choices. Evaluation of these policy changes are needed.

Children and adolescents consistently and substantially underestimate calories and sodium in QSR meals [150,151] and adolescents who visit QSR chains two or more times weekly are less likely to use menu labeling [152]. Therefore, research on physical activity calorie equivalent (PACE) labeling that conveys minutes or miles required to expend energy consumed at restaurants may be a promising strategy for children and adolescents. About 40 percent of parents are likely to encourage their child to be physically active when shown PACE labeling [153]. Restaurant owners could partner with researchers to test the effectiveness of PACE labeling on young customers’ purchases with special attention to minimize any unintended consequences that may lead to disordered eating behaviors. Finally, restaurants could fund future research to explore how *proximity* and the order of choices can promote healthy options at point-of-choice and point-of-purchase. Evaluations are also needed to examine changes in energy selected and unanticipated compensatory behaviors that may neutralize healthy behaviors [154].

### Study Strengths and Limitations

This study is the first to apply a novel framework that takes a comprehensive approach to identify, categorize and examine the available evidence for the restaurant sector to create healthy environments for customers. We did not have access to proprietary restaurant-industry data that may have provided insights into business-related decisions. Moreover, several factors that may influence restaurant decisions were not examined. Children have favorite options before they arrive at restaurants, parents allow their children to select their own meals, adults share their meals with children, and restaurant staff who interact with young people and parents may not prompt healthy choices [155,156]. One′s peers influence restaurant ordering even when calorie labelling is used [157]. Future evaluations are needed to explore the perceptions of diverse stakeholders related to acceptability, feasibility and profitability of using marketing-mix and nudge strategies across different interventions [158,159]; to examine the extent and effectiveness of using these combined strategies in food-retail settings, schools and worksites; and to identify factors that will facilitate consumers’ use of restaurant menu labeling [160]. 

## 5. Conclusions

The U.S. restaurant sector has many underutilized opportunities to combine marketing-mix and nudge strategies to promote healthy food environments. This study tested a novel marketing-mix and choice-architecture framework to evaluate the nature and extent of voluntary strategies used by U.S. chain and non-chain restaurants to promote healthy food environments for customers. This framework could serve as a useful planning tool for restaurant owners and managers, as well as an evaluation approach for other public- and private-sector stakeholders in the U.S. and other countries to monitor and evaluate progress of the restaurant sector to meet healthy targets. 

## Figures and Tables

**Table 1:** Marketing-mix and choice-architecture framework used to evaluate U.S. restaurant-sector progress to promote healthy food environments for American children, adolescents and parents, 2006–2016.

Category	Strategy	Performance Metrics
Voluntary changes made to the ***properties*** of the restaurant environment and/or food, beverage and meal products served and sold in the restaurant environment to influence customers’ purchasing and consumption behaviors	**1. Place** Change the internal setting (i.e., lighting or visual cues) to influence customers’ expectations about the ambience or atmosphere to highlight food and beverage products that support healthy dietary guidelines *.	• Restaurant has used lighting or visual cues to create an ambience or atmosphere that highlights food and beverage products that support healthy dietary guidelines and a healthy food and eating environment.
	**2. Profile** Change the nutritional profile, quality, smell, taste, texture and flavor of food and beverage products that meet recommended nutrient targets to support healthy dietary guidelines *.	• Restaurant has reformulated or developed new products to improve the nutritional profile, quality, smell, taste, texture and flavor of food and beverage products that meet recommended nutrient targets to support healthy dietary guidelines. • Restaurant offers entrees, value and bundled meals with side dishes that meet recommended nutrient targets for energy (≤600 calories/meal for children and ≤700 calories/meal for teens and adults), fat (≤35% total calories), saturated fat (≤10% total calories), added sugars (≤35% total calories) and sodium (≤210 mg to 410 mg/meal item).
	**3. Portion** Reduce and/or standardize the portion size of food and beverage products that meet recommended nutrient targets to influence customers’ expectations about single servings and appropriate portions to support healthy dietary guidelines *.	• Restaurant has reduced and/or standardized the portion size of food and beverage products that meet recommended nutrient targets for energy (≤600 calories/meal for children and ≤700 calories/meal for adolescents and adults), fat (≤35% calories/item), saturated fat (≤10% calories/item), sugar (≤35% calories/item) and sodium (≤210 milligrams/item to 450 milligrams/item).
	**4. Pricing** Use pricing strategies (i.e., proportionate pricing for smaller portions and limiting price promotions on large portions) to increase sales and revenue for products that meet recommended nutrient targets to support healthy dietary guidelines *.	• Restaurant has used pricing strategies to promote smaller portions that are competitively priced compared to energy-dense and nutrient-poor options sold in larger portions and package sizes. • Restaurant has tracked sales and revenue for smaller-portion products that meet recommended nutrient targets to support healthy dietary guidelines.
	**5. Promotion** Use responsible food and beverage marketing practices (i.e., colorful packaging for smaller portions; changing the name, appearance of food or beverage product, appeal and attractiveness of products) that meet recommended nutrient targets to support healthy dietary guidelines *.	• Restaurant has implemented and enforced a policy to use responsible food and beverage marketing practices to promote products that meet healthy dietary guidelines to children, adolescents and parents. • Restaurant has used menu design principles (i.e., graphics and placement) to emphasize fresh, seasonal, and minimally processed food and beverage products for all customers. • Restaurant has implemented and enforced a policy to restrict the promotion of high fat, sugary and salty food and beverage products to young people through television advertising, toy premiums, licensed media characters, celebrity endorsement, mobile and digital marketing.
Voluntary changes made to the ***placement*** of food, beverage and meal products served and sold in the restaurant environment to influence customers’ purchasing and consumption behaviors	**6. Healthy Default Picks** Use environmental cues that are convenient, accepted and expected to socially normalize healthy default choices for side dishes and beverages for children, adolescents and parents.	• Restaurant has implemented and enforced a policy to offer healthy default side dishes (e.g., fruits and vegetables) with bundled meals; healthy beverages (e.g., low-fat or non-fat milk, 100% juice and water); and whole grains with all meals sold to children, adolescents and parents.
	**7. Priming or Prompting** Use information (e.g., menu labeling and contextual information) to help customers make healthy decisions at point-of-choice and point-of-purchase.	• Restaurant has fully implemented and complied with the Food and Drug Administration’s menu-labeling regulations prior to the mandatory start date in May 2018 to help inform customers’ healthy choice purchases.
	**8. Proximity** Place healthy choices at eye level and physically closer to customers at point-of-choice and point-of-purchase.	• Restaurant has placed fruits, vegetables, salads and whole grains closer to customers’ point-of-choice (i.e., buffet lines) and point-of-purchase (cash register) locations.

* Dietary Guidelines for Americans 2015–2020 and other expert recommendations (i.e., USDA′s Smart Snacks in School Standards, School Meal Standards, and Healthy Eating Research Healthy Beverage Guidelines.

**Table 2:** Approach for acquiring and organizing the evidence to evaluate U.S. restaurant-sector progress, December 2006–January 2017.

The LEAD Principles (i.e., *Locate*, *Evaluate* and *Assemble* Evidence to Inform *Decisions*) were Used to Establish the Search Strategy	
***Search terms*** Search syntax: “Restaurant,” “Food” (MeSH terms) OR diet OR nutrition OR health OR sustainable OR sustainability AND “diet quality” (MeSH terms) OR calories OR fat OR salt OR sodium OR sugar OR portion size OR promotion OR advertising OR marketing OR priming OR proximity OR prompting OR policy OR guideline OR standard OR regulation. ***Inclusion criteria*** Peer-reviewed journal articles that were descriptive (i.e., cross-sectional or longitudinal), experimental studies, and systematic reviews conducted between 2006 and early 2017 for U.S. restaurants that reported on results for one or more of eight nudge strategies used in the marketing-mix and choice-architecture framework (Table 1). Gray literature reports, and media stories or news releases regarding restaurant use of one of eight nudge strategies between 1 January 2006 and 30 January 2017. ***Exclusion criteria*** Non-U.S. information sources. Interventions or strategies that did not align with the marketing-mix or choice-architecture strategies in the framework. Individual restaurant company documents or proprietary restaurant sector-documents that were not available in the public domain. ***Evidence selection criteria*** Qualitative-research criteria (i.e., data relevance, research-design quality, professional judgment, contextual relevance and credibility by data verification). Investigator and data triangulation were used for evidence convergence.	
**LOCATE Evidence**	**A literature review was conducted between 1 January 2006 and 30 January 2017 using the following sources:** Eight electronic databases (i.e., ABI/INFORM, Business Source Complete, CINAHL, Communication & Mass Media, Health Source, Medline, PsycInfo and PubMed) Websites of the top 20 limited-service, fast-casual and full-service U.S. restaurant companies Studies or reports released by government, industry, NGOs, private foundations and academic institutions Media stories, press and news releases
**EVALUATE Evidence**	**The investigators selected and categorized evidence sources (*n* = 84) into three evidence tables described below.** Table S1 summarizes the peer-reviewed articles (*n* = 49) and Table S2 summarizes the gray-literature reports (*n* = 18) compiled to evaluate the U.S. restaurant-sector progress using the eight marketing-mix or nudge strategies: ➢ Lead author, year, reference number ➢ Data collection period and study design (e.g., *cross-sectional, longitudinal or descriptive*). ➢ Data sources (e.g., *Menustat database, USDA nutrient database, University of Minnesota’s food and nutrient database, What We Eat in America survey, and information from restaurant websites, menus or customers’ receipts*). ➢ Assessment tools (e.g., Healthy Eating Index 2005 and 2010 or the Children’s Menu Assessment). ➢ Dietary standards (e.g., Dietary Guidelines for Americans or the NRA nutrition criteria). ➢ Restaurant chains sampled and study location (e.g., *city, state or national*). ➢ Results and outcomes measured. Table S3 summarizes relevant media stories or press or news releases (*n* = 27) organized chronologically (2006 through early 2017): ➢ Source and date ➢ Title ➢ Description **Criteria used to assign a progress score is based on the number of actions taken toward the specific performance metric:** **none**: no evidence of any action **limited**: evidence of 1–2 specific + actions that exceed − actions **some**: evidence of 3−4 specific + actions that exceed − actions **extensive**: evidence of 5 or more + actions that exceed – actions
**ASSEMBLE Evidence**	The marketing-mix and choice-architecture framework was used to organize the evidence based on the 8 strategies and 12 performance metrics. The strategies included: (1) place, (2) profile, (3) portion, (4) pricing, (5) promotion, (6) healthy default picks, (7) priming or prompting, and (8) proximity.
**Inform Decisions**	Table 4 summarizes actions that individual chain and non-chain restaurants (i.e., QSR, FCR and FSR) and the restaurant sector could take to promote healthy food environments for American customers. Actions are drawn from the recommendations issued by 16 authoritative bodies: ➢ Institute of Medicine′s Committee on Food Marketing to Children and Youth (2006) ➢ Food and Drug Administration′s Keystone Center′s Forum on Away-From-Home Foods (2006) ➢ Federal Trade Commission′s first evaluation report of industry marketing practices to children and teens (2008) ➢ White House Task Force on Childhood Obesity (2010) ➢ National Salt Reduction Initiative (2010) ➢ National Restaurant Menu Labeling legislation (2010) ➢ Federal Interagency Working Group on Foods Marketed to Children (2011) ➢ National Restaurant Association Kids LiveWell program (2011) ➢ Institute of Medicine′s Committee on Accelerating Progress to Prevent Obesity (2012) ➢ Federal Trade Commission’s second evaluation report of industry marketing practices to children and teens (2012) ➢ National Institutes of Health and RAND Corporation’s expert panel report for restaurants (2013) ➢ Culinary Institute of America and President and Fellows of Harvard College release Menus of Change Principles for restaurants (2013) ➢ Children′s Food and Beverage Advertising Initiative′s (CFBAI′s) uniform criteria for restaurant members (2014) ➢ Robert Wood Johnson Foundation′s Responsible Food Marketing to Children and Adolescents (2015) ➢ Dietary Guidelines Advisory Committee Report (2015) ➢ FDA’s final labeling guidelines for chain restaurants selling away-from-home foods (2016)

**Table 3:** U.S. restaurant categories, definitions and examples.

Category	Definition	Examples
Chain restaurants	Businesses operated at more than 20 locations under shared corporate ownership or franchising agreements in the United States (U.S.).	All restaurants featured below
Non-chain restaurants	Businesses that are owned and operated independently at fewer than 20 U.S. locations.	
Limited-service restaurants	Includes quick-serve restaurants (QSR) and fast-casual restaurants (FCR).	
Fast-food or quick-serve restaurants (QSR)	Restaurants with minimal service where food and beverages are ordered and received quickly.	Arby′s, Burger King, Carl′s Jr., Dairy Queen, KFC, McDonald′s, Popeyes, Sonic, Subway, Taco Bell and Wendy’s
Fast-casual restaurants (FCR)	Restaurants that offer limited table or self-service and upscale décor than LSRs, and higher-priced checks between $8/meal and $15/meal.	Au Bon Pain, Chipotle, Cosi Dominoes, Five Guys, Panera Bread, Qdoba and Starbucks
Full-service restaurant (FSR)	Restaurants that offer full table service, are family friendly, and offer entrée prices usually under $20 per person.	Applebees, Olive Garden and Silver Diner

**Table 4:** Recommendations issued by authoritative bodies for the U.S. restaurant sector to promote healthy food environments to American customers, 2006–2016.

Year	Authoritative Body
2006	IOM released an expert committee report on Food Marketing to Children and Youth
2006	FDA and the Keystone Center released a report of the interdisciplinary Forum on Away-From-Home Foods
2008	FTC released the first monitoring report on industry marketing practices to children and adolescents
2010	White House Task Force on Childhood Obesity released a multi-federal agency report to reverse obesity rates
2010	National Salt Reduction Initiative released sodium targets for the packaged and restaurant industries
2010	U.S. Congress passed the National Restaurant Menu Labeling Law (Section 4205 of Public Law 111–148 (H.R. 3590))
2011	Federal Interagency Working Group on Foods Marketed to Children released draft guidelines for healthy food marketing to children
2011	National Restaurant Association and Healthy Dining launched the *Kids Live Well* Program
2011	CBBB released the CFBAI′s uniform nutrition criteria for members including restaurant companies
2012	IOM released an expert committee report on Accelerating Progress to Prevent Childhood Obesity
2012	FTC released a second monitoring report on industry marketing practices to children and adolescents
2013	NIH and RAND Corporation′s expert panel released an expert report to establish restaurant standards
2013	Culinary Institute of America and President and Fellows of Harvard College released Menus of Change Principles for the restaurant sector
2015	RWJF’s Healthy Eating Research expert panel released recommendations for responsible food marketing to children
2015	Dietary Guidelines Advisory Committee Report released with specific recommendations for the restaurant sector
2016	FDA released the final labeling guidelines for chain restaurants selling away-from-home foods

Abbreviations: Children′s Food and Beverage Initiative (CFBAI), Council of the Better Business Bureaus (CBBB), Food and Drug Administration (FDA), Federal Trade Commission (FTC), Institute of Medicine (IOM), National Institutes of Health (NIH), National Restaurant Association (NRA), Research and Development (RAND) Corporation and Robert Wood Johnson Foundation (RWJF). Note: In 2016, the IOM was renamed the Health and Medicine Division (HMD) of the National Academies of Sciences, Engineering and Medicine.

**Table 5:** Progress scores and priority actions suggested for the U.S. restaurant sector to nudge American children, adolescents and parents toward healthy food environments.

#	Strategy	Progress Score (2006–2016)	Justification	Priority Actions for Chain and Non-Chain Restaurants
1	Place	None	**No evidence to evaluate progress**	• Create an ambience or atmosphere that encourages healthy food and beverage choices and consumption • Use lighting and visual cues to make healthy choices appealing to children, teens and parents
2	Profile	Limited	***N* = 32 studies or gray-literature reports** • 18 of 25 studies showed either a *modest calorie reduction* (+), *no calorie reduction* (−) *or no meaningful changes in fat, saturated fat and added sugars* (−) • 9 studies or reports showed either a *modest reduction* (+), *no reduction* (−) *or an increase in the sodium content of menu offerings* (−) • 3 studies or reports showed that *1–13 percent of meals met healthy dietary criteria recommended by authoritative bodies* (−)	• Reformulate 100% of entrees, value meals, side dishes and desserts for children, teens and parents that meet recommended nutrition targets including: • calories: children′s meals (≤600 kcal/meal) and teen and adult meals (≤700 kcal/meal) • fat (≤35 percent calories) and saturated fat (≤10 percent calories) • added sugars (≤35 percent calories) • sodium (≤210 mg to 450 mg)
3	Portion	Some	***N* = 4 studies** • 2 studies showed that a reduction of meal or side dish portions led to fewer calories purchased or consumed by children (+) • 2 studies showed that restaurant owners who participated in healthy restaurant programs were receptive to changes including reduced portions for children’s meals (+)	• Reduce and standardize 100% of portions for all entrees, value meals, side dishes and desserts for children, teens and parents that meet recommended nutrition targets including: • calories: children’s meals (≤600 kcal/meal) an teen and adult meals (≤700 kcal/meal) • fat (≤35 percent calories), saturated fat (≤10 percent calories) • added sugars (≤35 percent calories) • sodium (≤210 mg to 450 mg)
4	Pricing	Limited	***N* = 4 studies or reports** • The studies showed that increasing the quality and cost of healthy children′s meals did not affect restaurant revenues or customer orders (+) • No evidence to show progress made by leading QSR, FCR or FSR chains to reduce price promotions on large portions and package sizes (−) and use of competitive pricing to encourage healthy meals (−) • Changes were made by one FSR chain to reduce calories in children’s meals but total energy still exceeded the recommended ≤600 calories/meal (−)	• Use pricing strategies to competitively price healthy products that meet recommended nutrition criteria and reduce price promotions on larger portions and package sizes to remove the financial incentive of choosing larger options
5	Promotion	Limited	***N* = 12 studies, gray-literature reports and media releases** • In 2011, the NRA launched the Kids LiveWell Program that established nutrition criteria for children’s meals (+) but no guidelines for proportion of meals (e.g., 50%) that should meet the criteria for participating restaurants (−) • Between 2012 and 2015, McDonald′s made implemented and achieved several pledges to improve the nutritional quality and marketing of children′s meals (+) • Racially diverse teens were disproportionately targeted by restaurant advertising for unhealthy choices (−) • Restaurants continued to use marketing practices not covered by voluntary pledges to promote foods and beverages that did not meet nutrition targets including toy premiums (−), licensed media characters (−), brand mascots (−) and celebrities (−)	• Adopt responsible food and beverage marketing policies and practices that only promote healthy menu offerings • Restrict the promotion of HFSS foods and beverages to young people through all forms of integrated marketing communications especially TV advertising, toy premiums, licensed media characters, celebrity endorsement, mobile and digital marketing.
6	Healthy Default Picks	Limited	***N* = 3 studies, gray-literature reports and media releases** • One study found children and teens would select fruits and vegetables as healthy default choices (+) but 60–70 percent of 20 restaurants surveyed did not offer healthy defaults (−) • The Walt Disney Company study documented that 48-66 percent of customers accepted healthy default choices offered by their restaurants (+) • One study found only one of 50 restaurant chains offered healthy defaults in 2013 (−) • Between 2014–2016, 10 restaurant chains announced setting healthy default beverages (e.g., water, low-fat milk) for children′s meals to replace sugar-sweetened beverages (+) • NFVA found that the restaurant sector made limited progress to establish fruits and vegetables as healthy default choices 2005–2015 (−)	• Implement policies that offer healthy defaults such as: (1) Replace energy-dense side dishes (e.g., fries and chips) with healthy picks (e.g., fruits and vegetables); (2) Replace high-calorie beverages (e.g., soda) with healthy beverages (e.g., low-fat or non-fat milk, 100% juice and water); and (3) Replace unlimited self-serve SSB refills with free water refills.
7	Priming or Prompting	Limited	***N* = 4 studies** • One study in Washington State found a reduction of 19 calories/item and 73 calories/item, respectively before and after county menu labeling took effect. These restaurants exceeded the calorie and saturated fat targets (+,−) • One study found that <two-thirds of 400 restaurants offered menu labeling before 2010 and restaurants not providing menu labeling had higher fat and saturated fat content (−) • No changes in calories or sodium of children′s meals in 2011 (−) • Between 2012–2014, restaurants that had voluntarily implemented menu labeling offered lower-calorie items compared to restaurants that did not offer labeling (+)	• Implement and comply fully with the FDA′s menu labeling regulations effective May 2018 to help inform customers’ healthy choice purchases • Partner with researchers to test effectiveness of PACE labeling
8	Proximity	None	**No evidence to evaluate progress**	• Place healthy food and beverage choices at eye level and physically closer to customers at point-of-purchase and point-of-choice

Abbreviations: FCR (fast-casual restaurants), FDA (Food and Drug Administration), FSR (full-service restaurants), HFSS (high-fat, sugar and sodium) kcal (kilocalories), LSR (limited-service restaurants), NRA (National Restaurant Association), PACE (Physical Activity Calorie Equivalent) labeling QSR (quick-service restaurants), TV (television).

## Supplementary Materials

The following are available online at www.mdpi.com/1660-4601/14/7/760/s1. Table S1. Peer-reviewed articles used to evaluate the U.S. restaurant industry′s progress to create healthy food environments, 1 January 2006–30 January 2017, Table S2. Gray-literature reports used to evaluate the U.S. restaurant industry′s progress to create healthy food environments, 1 January 2006–30 January 2017, Table S3. Media stories or news releases used to evaluate the U.S. restaurant industry’s progress to create healthy food environments, 1 January 2006–30 January 2017.
