# Supplementary material for: Progress Evaluation for the Restaurant Industry Assessed by a Voluntary Marketing-Mix and Choice-Architecture Framework That Offers Strategies to Nudge American Customers toward Healthy Food Environments, 2006–2017
Source: Int J Environ Res Public Health. 2017 Jul 12;14(7):760. doi: 10.3390/ijerph14070760 (PMC5551198; doi:10.3390/ijerph14070760)
Supplement: Supplementary file 1 [file ijerph-14-00760-s001.pdf]

Supplemental Table 1 Peer-reviewed articles used to evaluate the U.S. restaurant industry's progress to create healthy food environments for American customers, 1 January 2006–30 January 2017

**Lead Author, Year**	**Data collection period (years)**<br>**Study design**	**Data sources**<br>**Assessment tools**	**Restaurants sampled**<br>**Study location**	**Results and outcomes measured**
**1. PLACE** ***(Improve the ambience or atmosphere to promote healthy restaurant food and beverage offerings)*** (n=0)				
No articles identified				
**2. PROFILE** ***(Restaurants change the nutrient composition to provide food and beverage offerings that promote a healthy diet)*** (n=25)				
Auchincloss et al., 2014 [1]	2011<br><br>Descriptive<br><br>Cross-sectional	Restaurant websites and print menus<br><br>Dietary Guidelines for Americans (DGA) 2010	22 quick-service restaurants (QSRs), fast-casual restaurants (FCRs) and full-service restaurant (FSR) chains including:<br>Denny's<br>Friendly's<br>IHOP<br>Pizza Hut<br>Applebee's Neighborhood Grill & Bar<br>Bertucci's Italian Restaurant<br>California Pizza Kitchen<br>Champps Americana<br>Chili's Grill & Bar<br>Famous Dave's Legendary Pit Bar-B-Que<br>Hard Rock Café<br>Houlihan's<br>LongHorn Steakhouse<br>Olive Garden<br>Red Lobster<br>Ruby Tuesday	The average calorie content of *à la carte* entrees and appetizers was 800 calories and did not meet the healthier criteria for calories 47% of the time.<br><br>About 30% of *à la carte* entrees exceeded the daily recommended values for saturated fat and sodium and about 20% met the recommended dietary fiber content.

			T.G.I. Friday's<br>Ted's Montana Grill<br>The Capital Grille<br>Morton's and The Steakhouse	
Ahuja et al., 2015 [2]	2010–2013<br><br>Descriptive<br><br>Cross-sectional	What We Eat In America (2007-2008) data used the FDA-defined sodium limits and the Healthy Eating Index 2010 "optimal" sodium levels	4 QSR chains including:<br>McDonald's<br>Burger King<br>Dominos<br>Pizza Hut<br>Local Chinese restaurants (unidentified)	A total of 88% (29 of 33 sampled restaurant foods) exceeded the FDA recommendations for "healthy" limits for sodium.
Bauer et al., 2012 [3]	2006–2010 (period of interest) although trends in the energy content of meal items were assessed between 1997/1998 and 2009/2010<br><br>Descriptive<br><br>Longitudinal	University of Minnesota Nutrition Coordinating Center's Food and Nutrient Database	8 QSR chains including:<br>McDonald's<br>Burger King<br>Wendy's<br>Taco Bell<br>Kentucky Fried Chicken<br>Arby's<br>Jack in the Box<br>Dairy Queen	General menu, entrée, and beverage median energy content did not differ between 2006 and 2010, and side-items decreased in caloric content. The energy content of condiments and desserts increased over the review period.
Bleich et al., 2015 [4]	2012–2013<br><br>Descriptive<br><br>Longitudinal	MenuStat Database 2012–2013	66 restaurant chains (not specified)	Menu items (n= 19,417) offered in 2012 or 2013 did not have any meaningful calorie-reduction changes. Newly introduced items in 2013 had lower calories (-56) than similar 2012 items. Calorie declines were among new main-course items (-10%, -67 calories), new beverages (-8%, -26 calories) and children's menu items (-20%, -46 calories).
Bleich et al., 2016 [5]	2012–2014<br><br>Descriptive<br><br>Longitudinal	Menustat Database 2012–2014	66 restaurant chains (not specified)	Calories declined in newly introduced menu items from 2012 to 2013 (-71), and from 2012 to 2014 (-69) (n = 23,066 total menu items). New main-course items decreased by 30

				calories (from 85 to 55 calories) (2013 to 2015). Although these items decreased in mean calories, they were higher in calories than items offered all three years. Items offered all three years had no significant difference in mean calories.
Bruemmer et al., 2012 [6]	May–Jun 2009 (pre)<br>May–Jun 2010 (post)<br><br>Intervention<br><br>Cross-sectional	Restaurant chain menus and websites compared to the DGA 2005	37 restaurant chains in King County, Washington including:<br>LSRs (n=26) and FSRs (n=11)	Energy contents were lower at FSRs (-7%, -73 calories) and LSRs (-3%, -19 calories) from 2009 to 2010, However, nutrition targets were exceeded for calories (56%), saturated fat (77%) and sodium (89%) at all restaurant locations.
Dumanovsky et al., 2009 [7]	Mar–Jun 2007<br><br>Descriptive<br><br>Cross-sectional	Customer receipts and surveys<br><br>Restaurant website information used to obtain caloric content of meals purchased	11 QSR chains in NYC including:<br>McDonald's<br>Burger King<br>Wendy's<br>Subway<br>Au Bon Pain<br>KFC<br>Popeye's<br>Domino's<br>Papa John's<br>Pizza Hut<br>Taco Bell	7,318 customer receipts were obtained from 167 FSR locations in NYC between lunch hours of 12 and 2 pm for baseline data prior to the mandatory national restaurant menu labeling implementation.<br><br>Customers purchased an average of 827 calories. Excluding single-item purchases, the average calorie content purchased rose to 961 calories. Chicken LSR chains averaged the highest calories purchased (931 calories), followed by Tex-Mex (Taco Bell; 900 calories), and burger chains (857 calories).<br><br>One in three customers purchased >1000 calories.

Hearst et al., 2013 [8]	2009–2010 (period of interest) although the period of 1997–1998 was assessed<br><br>Descriptive<br><br>Longitudinal	University of Minnesota Nutrition Coordinating Center Food and Nutrient Database<br><br>Healthy Eating Index (HEI) 2005	8 QSR chains including:<br>McDonald's<br>Burger King<br>Wendy's<br>Taco Bell<br>KFC<br>Arby's<br>Jack in the Box<br>Dairy Queen	HEI scores increased in 6 of the 8 QSR chains but the overall nutrition quality was still poor in menu offerings.
Hill et al., 2013 [9]	2013<br><br>Descriptive<br><br>Cross-sectional	Nutrition Environment Measures Survey for Restaurants<br><br>Children's Menu Assessment (CMA) tool	137 restaurants in the rural Appalachian region of south central Virginia and north central North Carolina	CMA scores were significantly lower in African-American block groups than white or mixed-race block groups.<br><br>Only 11% (n=15 out of 137 children's menus) included one or more healthy entrees.
Jacobson et al., 2013 [10]	2006-2011 (period of interest) although data assessed from 2005–2011<br><br>Descriptive<br><br>Longitudinal	Restaurant website data compared to the DGA 2010	16 QSR chains including:<br>Arby's<br>Au Bon Pain<br>Blimpie<br>Burger King<br>Chick-fil-A<br>Domino's Pizza<br>Hardee's<br>Jack in the Box<br>KFC<br>Little Caesars Pizza<br>McDonald's<br>Panera Bread<br>Papa John's Pizza<br>Pizza Hut<br>Subway<br>Wendy's	The average sodium content of 78 restaurant foods at 16 QSR chains increased by 2.6% from 2005 to 2011.
Jarlenski et al., 2016 [11]	2012 to 2014	Menustat Database 2012–2014	37 QSR and FCR chains (not specified)	From 2012 to 2014, only minor declines in calorie content (22-

	Descriptive<br><br>Longitudinal			calorie reduction) was observed among food items across 11,737 menu items assessed for changes in macronutrient composition (non-sugar carbohydrates, sugar, unsaturated fat, saturated fat and protein).<br><br>Over this time period, beverages revealed an increase by 46 calories, newly introduced main course items reduced by 59 calories, and newly introduced dessert items showed an increase by 90 calories (represented by an increase of 57 calories from added sugar).
Kirkpatrick et al., 2013 [12]	2006<br><br>Descriptive<br><br>Cross-sectional	Restaurant websites menus coded using Food and Nutrient Database for Dietary Studies and MyPyramid Equivalents Database<br><br>HEI-2005 score	5 QSR chains including:<br>Burger King<br>McDonald's<br>Subway<br>Taco Bell<br>Wendy's	Full menus at QSR chains scored lower than 50/100 points on the HEI-2005. Children's menus scored 10 points higher on average, and items marketed as healthy or nutritious scored 17 points higher compared to full menus.
Krukowski et al., 2011 [13]	2009–2010<br><br>Descriptive<br><br>Cross-sectional	Restaurant provided children's menu<br><br>Children's Menu Assessment tool	130 local and QSR chains located within 20 miles of Little Rock, Arkansas	Healthy options were less prevalent for children's menus (13% had a healthy entrée) and parents were not provided sufficient information to make healthy menu choices.
Moran et al., 2017 [14]	2012–2015<br><br>Descriptive	MenuStat Database<br><br>Analysis of children's menu items (n=4,016)	45 U.S. restaurant chains (not specified) including:<br><br>26 national<br>19 regional<br>23 QSR<br>19 FSR	From 2012 to 2014, calories in beverages offered with children's menus increased significantly by 11 calories. From 2012 to 2015, no significant changes were observed for calories in

			6 FCR	beverages, total calories, sodium or saturated fat in children's menu food offerings. Restaurants that participated in the Kids LiveWell program (n=15) significantly reduced children's entrée calories between 2012 and 2014 (by 40 calories/meal) compared to non-participating restaurants but this change did not persist for the 2012 to 2015 period.
O'Donnell et al., 2008 [15]	2007<br><br>Descriptive<br><br>Cross-sectional	USDA database<br>Nutrient Analysis Protocol from the National School Lunch Program (NSLP)	10 QSR chains in the Houston, Texas area including:<br>Arby's<br>Burger King<br>Chick-fil-A<br>KFC<br>McDonald's<br>Sonic<br>Subway<br>Taco Bell<br>Wendy's<br>Whataburger	Only 3% of possible children's meal combinations met the 2007 NSLP nutrient guidelines.
Rudelt et al., 2014 [16]	2006-2010 (period of interest) although trends in sodium content were assessed between 1997/1998 and 2009/2010<br><br>Descriptive<br><br>Longitudinal	University of Minnesota Nutrition Coordinating Center Food and Nutrient Database	8 QSR chains including:<br>McDonald's<br>Burger King<br>Wendy's<br>Taco Bell<br>KFC<br>Arby's<br>Jack in the Box<br>Dairy Queen	Between 2006 and 2010, no meaningful reduction in mean sodium content of the menu offerings (e.g., lunch and dinner entrees and side dishes) was observed for the eight QSR chains analyzed.
Schoffman et al., 2016 [17]	2014<br><br>Descriptive<br><br>Cross-sectional	MenuStat Database	34 QSR chains including:<br>White Castle<br>Krystal<br>Subway<br>Wienerschnitzel<br>Taco Bell<br>In-N-Out Burger	A total of 3,193 entrées were analyzed for 34 QSR and 28 FCR chains (total = 62 restaurants).<br><br>FCRs provided significantly more calories per entrée (760

			A&W<br>Del Taco<br>McDonald's<br>Chick-Fil-A<br>Taco Bueno<br>Arby's<br>Taco John's<br>Burger King<br>Hardee's<br>Wendy's<br>Charley's Grilled Subs<br>Tropical Smoothie Café<br>Jack in the Box<br>Dairy Queen<br>KFC<br>El Pollo Loco<br>Checker's Drive-In/Rally's<br>Sonic<br>Church's Chicken<br>Carl's Jr.<br>Fazoli's<br>Quiznos<br>Jimmy John's<br>Whataburger<br>Bojangle's<br>Popeye's<br>Taco Cabana<br>Long John Silvers<br><br>28 FCR chains including:<br>Panda Express<br>Steak 'N Shake<br>Einstein Brothers<br>Bruegger's Bagels<br>Five Guys<br>Au Bon Pain<br>Panera Bread<br>Noodles & Company<br>Cosi<br>Qdoba	kcal ± 301 kcal) than QSR entrées (561 kcal ± 268).<br><br>QSRs provided significantly more entrées in the lower-calorie categories (≤ 500 calories/item) and FCRs provided more entrées in the higher-calorie categories (≥ 751 calories/item).

			Schlotzsky's<br>Potbelly's Sandwich Works<br>Chipotle<br>Corner Bakery Café<br>Pollo Tropical<br>Culver's<br>McAlister's Deli<br>Jason's Deli<br>Moe's Southwest Grill<br>Smashburger<br>Dickey's Barbecue Pit<br>Togo's Eatery/Sandwiches<br>Baja Fresh<br>Zaxby's<br>Firehouse Subs<br>Captain D's<br>Pei Wei<br>Boston Market	
Serrano and Jedda, 2009 [18]	Jan 2006 (period of interest) data were also collected in December 2005<br><br>Descriptive<br><br>Cross-sectional	Children's meals from non-fast food restaurants were ordered, weighed, and underwent nutritional analysis. Children's meal offerings from fast food restaurants were recorded and nutrient content was obtained from company websites.	Blacksburg, VA<br><br>10 QSRs and 23 non-chain restaurants not identified	Non-fast food children's items contained higher percentages of calories from fat (40.5% vs. 37.5%), grams of fat (31.7g vs. 19.4), and saturated fat (11g vs. 5.8g) than the children's items offered at QSRs.<br><br>All metrics were significantly different except for calories from fat.
Sliwa et al., 2016 [19]	May 2014<br><br>Descriptive<br><br>Cross-sectional	Data were collected from the top 10 FSR and LSR restaurants in the U.S. from 2013 rankings. Menu screenshots were captured from restaurant websites for child menus.<br><br>Nutrition criteria aligned with the 2010 DGAs, expert recommendations, and criteria	10 QSR chains including:<br>Arby's<br>Burger King<br>Chik-Fil-A<br>Dairy Queen<br>Jack-in-the-Box<br>KFC<br>McDonald's<br>Sonic<br>Subway<br>Wendy's	The majority of selected QSR and FSR children's meal combinations (72%, 63%) met the calorie criteria of $\leq$ 600 kcal per meal.<br><br>Only 31.9% of children's combination meals met all four nutrition criteria ($\leq$ 600 kcal,

		endorsed by the National Restaurant Association.	10 LSR chains including:<br>Applebee's<br>Buffalo Wild Wings<br>Chili's<br>Denny's<br>IHOP<br>Olive Garden<br>Outback Steakhouse<br>Red Lobster<br>Red Robin<br>TGI Friday's	≤ 35% kcal from fat, ≤ 105 kcal from saturated fat and ≤ 770 mg of sodium).
Urban et al., 2011 [20]	Jan–Jun 2010<br><br>Descriptive<br><br>Cross-sectional	Restaurant provided menus	42 restaurant locations (n=21 LSRs and 21 FSRs) located around Boston, MA, Little Rock, AK and Lafayette, IN	Foods were ordered from restaurant locations and calorie content was compared to reported content using bomb calorimetry. Of the 269 items analyzed, the average calories per portion was 10 calories higher than the stated energy content.
Urban et al. 2013 [21]	Jun–Aug 2011<br><br>Descriptive<br><br>Cross-sectional	Used a random selection of restaurant foods within study location; meal energy content was analyzed using bomb calorimetry. The 5 most popular dinner entrées and accompanying side dishes from each restaurant were analyzed.	36 FSR non-chain restaurants located in Boston, MA<br><br>2 small (< 10 employees) and 2 large (≥ 10 employees)<br><br>FSRs were randomly chosen from the types of most common restaurant categories nationwide: Mexican, American, Chinese, Italian, Japanese, Thai, Indian, Greek, and Vietnamese	Of the 157 meals included in the final analysis, the average caloric content was 1,327 kcal. The energy content of American, Chinese, Indian, and Italian meals was greater than the mean; and Mexican, Greek, Thai, Vietnamese, and Japanese meals were less than the mean. Three quarters (75.2%) of individual meals contained at least 50% of the daily energy requirement for a 2,000 kcal/person/day intake.
Urban et al., 2014 [22]	2006–2013 (period of interest) although trends in saturated fat and sodium were assessed between 2000 and 2013	Nutrient composition of food items obtained from Wayback Machine Website	3 QSR chains (not specified)	Three items were compared across the time period on their sodium, saturated fat, and trans fat composition: fried potatoes,

	Descriptive<br><br>Longitudinal	DGA 2010		cheeseburgers and grilled chicken sandwiches. Fried potatoes had trans-fat declines to undetectable levels around 2008-2009. Overall, there was no consistent downward trend in sodium, or saturated fat for the sample. Cheeseburgers were the main contributor of trans fat after 2009, as well as saturated fat which did not significantly change over the study period. Only large fried potatoes showed sharp reductions in saturated fat and trans-fat content over the study period.
Urban et al. 2016 [23]	2011–2014<br><br>Descriptive	5 non-chain restaurants from the 9 most popular cuisines were randomly selected in target cities. Within cuisines the three most popular entrees and accompanying side dishes were ordered and analyzed using bomb calorimetry. 21 large-chain restaurants that had at least one outlet in at least two of the regions were used for comparison; nutrient data was obtained from the Nutrient Database for Standard Reference from the USDA.	45 non-chain restaurants<br>21 large-chain restaurants (not specified)<br><br>San Francisco, CA<br>Boston, MA<br>Little Rock, AR	Among meals that were matched between non-chain and large-chain, large-chain meals contained on average 68 fewer calories. Non-chain restaurant meals contained an average of 1,205 calories, and 92% of non-chain meals contained over 570 calories (benchmark energy requirement for an adult woman).
Wu and Sturm, 2012 [24]	Feb–May 2010<br><br>Descriptive<br><br>Cross-sectional	Restaurant websites<br>Research Report for Foodservice InfoUSA<br><br>Chains available at Restaurants and Institutions top 400 list for 2009	400 restaurant chains	A total of 61% of restaurants provided complete nutrition information. Children’s menu items and beverages contained more fat, saturated fat, and carbohydrates than standard menu beverages.<br>Restaurants that made nutrition information accessible had

		Kids LiveWell and USDA nutrition standards		lower energy, fat, and sodium contents than those providing information only upon request.
Wu and Sturm, 2014 [25]	Feb–May 2010<br>Apr–May 2011<br><br>Descriptive<br><br>Cross-sectional	Restaurant's websites/email request<br><br>Research Report for Foodservice Info USA	213 restaurant chains	No significant changes in energy or sodium were noted one year after the federal mandatory menu-labeling law was passed in 2010.
**3. PORTION** ***(Restaurants reduce and standardize the meal portion size and make other changes to meet recommended nutrition criteria)*** (n=7)				
Cohen et al., 2016 [26]	2014<br><br>Descriptive	2014 MenuStat database<br><br>Polling of 15 member expert panel on portion recommendations for children	MenuStat data available for the top 200 U.S. restaurant chains	The expert panel concluded that children's *à la carte* items and entrees should not contain more than 300 calories, and side dishes and dessert should not contain more than 150 calories/serving. Actual portion size/calorie content recorded for 200 U.S. restaurant chains exceeded the recommended amounts in all food categories except for fruit and vegetables, which were 46% and 69% of the recommended calorie content, respectively.
Crixell et al., 2014 [27]	Spring 2010–2014<br><br>Descriptive post-intervention	The Best Food FITS intervention included six components:<br>A brand with a logo, new partner in health community with restaurant sector, collected and reviewed existing children's menus, requested restaurant owners and managers to change practices, collaborating to improve children's menus, and evaluation the intervention	17 restaurants in San Marcos, TX	Of 135 restaurants approached by the Texas Department of State Health Services to develop the Best Foods FITS intervention, 65 restaurants declined to participate and 17 restaurants made changes including 7 restaurants that agreed to create healthier children's menus and 10 restaurants (including one

				chain) that revised existing menus based on the Best Food FITS criteria. Twelve restaurants were still using the revised menus on a two-year follow up in 2014.
Escaron et al., 2016 [28]	Oct 2011–Sept 2012<br><br>Descriptive post-intervention	Specific nutrition criteria developed for program approval: entrees must contain ≤ 700 kcal and side dishes contain ≤ 300 kcal	7 unspecified restaurants in Waupaca, WI	Waupaca Eating Smart was a rural community-specific program developed to promote healthy choices at restaurants and supermarkets. Coalition members worked with local restaurants to increase the amount of healthy options available to customers.
Gase et al., 2014 [29]	Sept–Nov 2012<br><br>Descriptive	Semi-structured interviews with restaurant owners and qualitative thematic analysis	18 restaurant owners representing 350 restaurants in Los Angeles, CA<br><br>Restaurants not identified	Six themes emerged from the interviews:<br>(1) perceived customer demand is central to menu planning,<br>(2) many restaurants already offer multiple portion sizes,<br>(3) perceived logistical barriers to offering reduced size portions,<br>(4) concerns about declining revenues when offering reduced-size portions,<br>(5) healthy eating is the responsibility of the customer,<br>(6) desire to be a socially responsible industry leader.<br><br>Restaurant owners can engage effectively to reduce portion sizes of meals especially for children.
Gase et al. 2015 [30]	(Pre) July 2013<br>(Post) July 2014<br><br>Intervention	Restaurant provided menus	17 unspecified restaurants in Los Angeles, CA that participated in the Choose LA Restaurants Program	After participating in the Choose LA Restaurants Program, which required specific portion, children’s

	Cross-sectional			meals, and water specifications, 9 of 17 brands made changes to their menus to meet the portion size criteria. All 17 restaurants were in full compliance with the program after joining.
Serrano and Jedda, 2009 [18]	Jan 2006 (period of interest) data was also collected in December 2005<br><br>Descriptive	Children’s meals from non-fast food restaurants were ordered, weighed, and analyzed.<br><br>Children’s meals from LSRs were recorded and the nutrient content was obtained from companies’ websites.	10 LSR and 23 non-chain restaurants not identified in Blacksburg, VA	LSRs offered significantly smaller portion sizes than non-fast food restaurants (211.3g vs. 302.7g). There was a large difference in the range of meal weight sampled from 3 ounces to 29.9 ounces.
Wansink and Hanks, 2014 [31]	June–Aug 2011 (pre)<br>June–Aug 2012 (post)<br><br>Intervention	Restaurant transaction and nutrient data	30 McDonald’s Corporation franchises across the U.S.	Reducing the portion size of fries and changes made to side-dishes (e.g., including apple slices, no caramel sauce and fat-free chocolate milk) for children’s meal bundle led to a reduction in ~19% fewer calories (98 calories/meal bundle).
**4. PRICING** ***(Increase customers’ purchases and the restaurants’ revenue for healthy restaurant food and beverage choices)*** (n=4)				
Anzman-Frasca et al., 2015 [32]	Sept-Mar 2011-2012<br>Sep-Mar 2012-2013<br><br>Descriptive post-intervention	Silver Diner outlet data<br><br>Kids LiveWell eligibility and USDA database	13 Silver Diner FSRs in New England	Following a change in the children’s menu (more healthy options, healthy sides as default options) prices increased $0.79 for breakfast and $0.19 for non-breakfast pre to post, while revenue continued to increase post-implementation.<br>Healthy meals, fruit and/or vegetable sides, milk and juice orders all increased, while orders of fries and soda decreased. Calories consumed decreased (684.2 vs 621.2) for children accepting defaults and

				did not change for those not accepting defaults (935.0 vs. 942.9).
Anzman-Frasca et al., 2015 [33]	Pre: Sept–Mar 2011–2012<br>Sept–Mar 2012–2013<br>Post: Sept 2013–Mar 2014<br>Sept 2014–Mar 2015<br><br>Intervention	Silver Diner outlet data, Kids LiveWell eligibility and USDA database	13 Silver Diner FSRs in New England	Using a one- and two-year follow up to improvements in children's menu choices (e.g., expanding healthy options and designating healthy default side dishes), the percentage of healthy options chosen rose from 3 percent in the pre-implementation period to ≤ 43% in the two follow-up periods. The total annual revenue across all locations grew by 5.31% from 2013-2014, which was higher than the average growth in leading FSR chains.
Krukowski and West, 2013 [34]	2012<br><br>Descriptive	Children's Menu Assessment and restaurant children's menus	75 FSRs across the U.S.	All 75 restaurants had less healthful entrées provided on children's menus.<br>More than three quarters (77%; n=58) of restaurants sold at least one or more healthful entrees. Mean price of the more healthy entrees was not significantly different from the price of the less healthy entrees (e.g., $5.38 versus $5.27).
Peters et al., 2016 [35]	October 2009–2012<br><br>Descriptive	Restaurant menu data provided Researchers classified meals as "classic" or "healthy"	145 Walt Disney World restaurants	Prices were the same for both healthy and classic meals, and children accepted the default healthy side dishes 48% of time and healthy beverage 66% of time.<br><br>Improving the nutritional quality of children's meals did not affect the meal cost for customers.

**5. PROMOTION** ***(Use responsible marketing practices to promote healthy food and beverage offerings for children and adolescents)*** (n=10)				
Bernhardt et al., 2013 [36]	July 2009–Jun 2010<br><br>Descriptive	Total TV ad content was identified and TV ads for children's meals were compared with TV ads for adult meals (n=92 ads)<br><br>Content coding included visual and audio assessment of branding, toy premiums, movie tie-ins, and food depictions	25 national QSR chains	A majority of QSR children's meal ads that aired during the study period were attributable to McDonald's (70%) or Burger King (29%).<br><br>79% of 25,000 TV ad placements aired on just four channels (Cartoon Network, Nickelodeon, Disney XD, and Nicktoons).<br><br>Visual branding was more common in children's ads versus adult ads.<br><br>Toy premiums or giveaways were present in 69% of children's ads versus 1% of adult ads.<br><br>Movie tie-ins were present in 55% of children's ads versus 14% of adult ads.<br><br>Audio script for children's ads emphasized giveaways and movie tie-ins versus adult ads that emphasized food taste, price and portion size.<br><br>QSR chains are not adhering to voluntary pledges to focus on food products instead of toy premiums.

Bernhardt et al., 2014 [37]	Jul 2010–Jun 2011<br><br>Descriptive	Children ages 3-7 years (n=99)<br><br>Children's were exposed to two advertisements from Burger King and McDonald's.	Northern New England pediatric setting	After being shown images from the two restaurants' healthy food advertisements:<br>(1) 51% and 70% of children identified milk from McDonald's and Burger King, respectively;<br>(2) 80% mentioned apples when describing McDonald's but only 10% mentioned apples for Burger King.<br><br>Of the four healthy food images shown, only apples shown by McDonald's was communicated adequately. Representations of milk were inadequately communicated to preliterate children. The investigators concluded that the Burger King's depiction of apple slices misled the children.
Bernhardt et al., 2015 [38]	2010–2011<br><br>Observational	Children ages 3-7 years (n=100)<br><br>Children's were exposed to QSR chain TV ads randomly selected as either children's or adult ads, and were asked what they recalled which was checked with the TV ad transcripts for descriptors of foods and premiums/tie-ins.	Northern New England pediatric setting<br><br>TV ads from either Burger King and McDonald's chains	Children were less likely to recall food after viewing the child versus adult-targeted ads.<br><br>Children were more likely to recall premium/tie-ins more and unhealthy food options more frequently versus healthy food or beverage options (i.e., apples and milk).<br><br>QSR TV ads are not effectively communicating healthy menu choices to young children that they remember.

Elbel et al., 2015 [39]	2013–2014<br><br>Descriptive	Data collected on site<br>Nutrition standards determined by the New York City (NYC) Healthy Happy Meals Bill Requirements	3 LSR chains in NYC and New Jersey locations including:<br>Burger King<br>McDonald's<br>Wendy's	Following proposed legislation that required children's meals with toys to meet nutritional criteria, data were obtained on current nutritional status of children's combination meals.<br><br>A total of 35% of children ate the combination meals but 98% of meals did not meet the proposed criteria for at least one nutrient (51% exceeded limit for calories, 55% for sodium, 78% for calories from fat, 14% for saturated fat, and 49% for added sugars).<br><br>If all children's meals met the proposed criteria, there would be a 10% reduction in sodium, 9% in calories, and a 10% reduction in calories from fat.
Emond et al., 2016 [40]	2011<br><br>Descriptive	Parents (n=100) of children ages 3-7 reported child's TV viewing habits, frequency to LSR restaurants that participated in current child-directed marketing, the child's request to visit these restaurants, and the collection of toy premiums at these restaurants.	2 LSR chains including:<br>McDonalds and Burger King (Self-reported child frequenting and/or requesting to visit these restaurant chains)	More than half (54%) of children requested visits to one of the two fast food restaurants.<br><br>More than a quarter (29%) of children collected toy premiums from these restaurants, and commercial TV viewing was greater in both these groups than those that did not request visits or take toy premiums.
Kraak and Story, 2015 [41]	2006-2015 (period of interest) although sources from 2000-2015 were used.<br><br>Descriptive	Reviewed descriptive studies, published books, and grey-literature reports (n = 34) as well as media stories, news releases, and public testimony (n = 54) in the U.S. that measured prevalence of cartoon brand	QSRs that used brand mascots (i.e., McDonald's and Chuck E Cheese)	There was very limited progress in the use of mascots and media character marketing to children in the restaurant sector.<br>In 2011 the NRA's *Kids LiveWell* Program provided nutrition standards but no best-

		mascots and media characters used to market food and beverages to children < 12 years.		practice marketing guidelines for use of toy premiums, brand mascots, or media characters to promote meals. No participating CFBAI or Kids LiveWell pledged to ensure that ≤ 50% of meals sold to children and adolescents met the DGAs.
Ohri-Vachaspati et al., 2015 [42]	2010–2012<br><br>Descriptive	InfoUSA<br>Dun and Bradstreet	6,716 QSRs across the U.S.	Overall, 31.4% of QSRs used child-directed marketing inside or on the exterior and 20.8% used indoor signage for toy premiums. Use of any child-directed marketing decreased from 34.9% to 27.4% between 2010 and 2011 but rose to 32.6% in 2012. Use of indoor displays for toy premiums did not change significantly. Restaurants in a majority African-American communities, rural areas, and middle-income communities had higher odds of having restaurants with child-directed marketing than urban and high income communities.
Otten et al., 2012 [43]	Jul–Aug 2010 (Pre)<br>Aug and Nov 2010 (Post)<br><br>Description post-intervention	Santa Clara County Toy Ordinance: 5-6 nutrition standards<br><br>CMA tool	4 QSR chains located in Santa Clara County, CA including: 3 global chains and 1 local chain (not identified)	In August 2010, Santa Clara County, CA implemented an ordinance that prohibits the distribution of toy premiums with food and beverages that do not meet minimal nutrition criteria. 4 months after the ordinance was implemented, CMA scores increased 2.8-3.4 fold. Restaurants did not increase the number of healthy options, improve items through reformulation, or provide toys freely with meals post-

				ordinance. Recommended improvements included removing toy signage, establishing a healthier default, and removing toys completely.
Otten et al., 2014 [44]	Jan–Mar 2011 (pre 1)<br>Oct–Nov 2011 (pre 2)<br>Jan–Mar 2012 (post)<br><br>Descriptive post-intervention	Street intercept survey	2 QSR chains in California including:<br>McDonald's<br>Burger King	Following the approval of an ordinance banning provision of toy premiums with children's meals that did not meet specific nutrition criteria, both McDonald's and Burger King began selling the toy premiums for 10 cents, and 88% of sampled customers purchased the toy.<br>Nutrition criteria changed as McDonald's reduced the size of fries offered in meal bundles and added apple slices (-110 kcal, -6g fat, -130 mg sodium), stopped serving caramel sauce with its apple slices (-70 kcal), and offered fat free milk, replacing for 1% milk.<br>Burger King announced it would join the Kids *LiveWell* program, that soda and fries would no longer be served as a default side dish, and stopped serving caramel sauce with its apple slices (-45 kcal).
Powell et al., 2010 [45]	200–2008 (period of interest)<br>Data collected during the years 2003, 2005, and 2007<br><br>Descriptive<br><br>Longitudinal	Nielsen Media Research television ratings data<br><br>NHANES 2003-04, 2005-06, 2007-08 data	10 QSR chains including:<br>Arby's<br>Burger King<br>Domino's Pizza<br>KFC<br>McDonald's<br>Pizza Hut<br>Sonic	Fast food advertisers disproportionately targeted African-American (child or teen) viewers in all measured age groups (2-5, 6-11, and 12-17), with the total African American to White ratio increasing from 1.52 in 2003 to

			Subway<br>Taco Bell<br>Wendy's	1.63 in 2007. Exposure to LSR and FSR advertisements increased over the study period; in 2007 LSR advertisements were the most frequently viewed food ads for all 3 age groups. Children 2 to 5, 6-11, and 12-17 years saw on average, 2.4, 3 and 4 ads per day, respectively. Children of all ages were most frequently exposed to McDonald's ads, and while adolescents saw more ads per day than children, children <12 years viewed more McDonald's advertisements than adolescents. Burger King was the second leading advertiser of the 10 QSRs, followed by Subway.
**6. Healthy Default PICKS *(Restaurants use environmental cues to socially normalize healthy default food and beverage choices)*** (n=4)				
Anzman-Frasca et al., 2014 [46]	Apr 2013<br><br>Descriptive	Online restaurant menus<br><br>Items coded by researchers based on presence/amount of fruit and/or vegetables (F/V)<br><br>Survey conducted among (n = 1,178) children and teens ages 8-18 years	QSR (n =10) and FSR (n =10) chains including:<br>McDonalds<br>Subway<br>Starbucks Coffee<br>Burger King<br>Wendy's<br>Taco Bell<br>Dunkin' Donuts<br>Pizza Hut<br>KFC<br>Chick-fil-A<br>Applebee's Neighborhood Grill & Bar<br>Olive Garden	Of the 20 restaurants surveyed, 60% of QSRs and 70% of FSRs did not serve fruits or vegetables as default choices for side dishes.<br><br>95% of children's menus had at least one fruit or vegetable (F/V) side dish available. LSRs offered a range of 0-3 F/V items while FSRs offered a range of 2 -12 F/V items.<br><br>About one third (32.8%) of children reported they would be

			Chili's Grill and Bar<br>Red Lobster<br>IHOP<br>Denny's<br>Outback Steakhouse<br>Buffalo Wild Wings<br>Cracker Barrel<br>T.G.I. Friday's	somewhat or very unhappy (30.7% of teens), while 32.3% reported they would be somewhat or very happy (31.2% of teens) if the children's meal came with a fruit or vegetable instead of fries as the default choice that would result in a 170-calorie reduction.
Gase et al., 2015 [47]	(Pre) Jul 2013<br>(Post) Jul 2014<br><br>Descriptive post-intervention	Restaurant provided menus	17 unspecified restaurant chains in Los Angeles that participated in the Choose LA Restaurants Program	After participating in the Choose LA Restaurants Program, which required specific portion, children's meals, and water specifications, 9 of 17 restaurants changed their menus to meet the portion size criteria. Participating restaurants (n=17) fully complied with the program.
Henry and Borzekowski, 2015 [48]	Nov 2011–Jan 2012<br><br>Descriptive	Perceptions of mothers were explored for food choices and default meal options for children ages 3-8 years (n = 40)<br><br>Qualitative analysis of telephone interviews	Restaurants not reported	Mothers praised QSRs for offering healthier choices but voiced concerns about food quality.<br>Mothers reported that their children wanted to visit restaurants because of advertised toys and not food offerings.<br>Half (n=20) of mothers liked bundled meals if they could choose specific items.<br>Mothers reported that offering healthy defaults could eliminate battles, reduce forgetfulness and facilitate ordering.
Peters et al., 2016 [35]	October 2009–2012<br><br>Descriptive	Restaurant menu data provided<br>Researchers classified meals as "classic" or "healthy"	145 Walt Disney World restaurants	Children accepted the default of healthy sides 48% of the time and the beverage 66% of the

				time. This reduced the calories (21.4%), fat (43.9%), and sodium (43.4%) for the side dishes and beverages chosen by children.
**7. PRIMING or PROMPTING *(Restaurants provide menu labeling and contextual information to promote healthy food and beverage choices)*** (n=3)				
Bleich et al., 2015 [49]	2012–2014	MenuStat Database	66 restaurant chains<br>(not specified)	Mean calorie content of foods offered at restaurants participating in voluntary menu labeling had lower calories (139 less calories in 2012, 136 in 2013, and 139 in 2014) than restaurants that did not participate in voluntary menu labeling.
Bruemmer et al., 2012 [6]	May–Jun 2009 (pre)<br>May–Jun 2010 (post)<br><br>Intervention<br><br>Cross-sectional	Restaurant chain menus and websites compared to the DGA 2005	37 restaurant chains in King County, Washington including:<br>LSRs (n=26) and FSRs (n=11)	After 18 months of enacting the restaurant menu labeling law, energy contents were lower at FSRs (-7%, -73 calories) and LSRs (-3%, -19 calories) than 6 months after labeling was initiated. However, nutrition guidelines were exceeded for calories (56%), saturated fat (77%) and sodium (89%) at all restaurant locations.
Wu and Sturm, 2012 [24]	Feb–May 2010<br><br>Descriptive<br><br>Cross-sectional	Restaurant websites<br>Research Report for Foodservice InfoUSA<br><br>Chains available at *Restaurants and Institutions* top 400 list for 2009<br><br>Kids LiveWell and USDA nutrition standards	400 restaurant chains	A total of 61% of restaurants provided complete nutrition information. Children's menu items and beverages contained more fat, saturated fat, and carbohydrates than standard menu beverages.<br>Restaurants that made nutrition information accessible had lower energy, fat, and sodium contents than those providing information only upon request.

Wu and Sturm, 2014 [25]	Feb–May 2010<br>Apr–May 2011<br><br>Descriptive<br><br>Cross-sectional	Restaurant's websites/email request<br><br>Research Report for Foodservice Info USA	213 restaurant chains	No significant changes in energy or sodium were made by the restaurant chains examined one year after the federal mandatory menu-labeling law was passed in 2010.
**8. PROXIMITY** ***(Restaurants make the healthy choice physically closer at point-of-purchase)*** (n=0)				
No articles identified				

Abbreviations and Acronyms

Children's Menu Assessment (CMA), Dietary Guidelines for Americans (DGA), U.S. Food and Drug Administration (FDA), fruits and vegetables (F&V), fast-casual restaurants (FCR), full-service restaurants (FSR), Healthy Eating Index (HEI), kilocalories (kcal), Los Angeles (LA), National School Lunch Program (NSLP), New York City (NYC), quick-service restaurants (QSR), Texas (TX), U.S. Department of Agriculture (USDA), Virginia (VA), Washington (WA) and Wisconsin (WI).

References

---

1. Auchincloss AH, Leonberg BL, Glanz K, Bellitz S, Ricchezza A, Jervis A. Nutritional value of meals at full-service restaurant chains. *J Nutr Educ Behav*. **2014,** *46*, 75–81. http://dx.doi.org/10.1016/j.jneb.2013.10.008.
2. Ahuja JK, Wasswa-Kintu S, Haytowitz DB et al. Sodium content of popular commercially processed and restaurant foods in the United States. *Prev Med Rep*. **2015,** *2*, 962–967. http://dx.doi.org/10.1016/j.pmedr.2015.11.003.
3. Bauer KW, Hearst MO, Earnest AA, French SA, Oakes JM, Harnack LJ. Energy content of U.S. fast-food restaurant offerings: 14-year trends. *Am J Prev Med*. **2012,** *43*, 490–497. http://dx.doi.org/10.1016/j.amepre.2012.06.03.
4. Bleich SN, Wolfson JA, Jarlenski MP. Calorie changes in chain restaurant menu items: implications for obesity and evaluations of menu labeling. *Am J Prev Med*. **2015,** *48*, 70–75. http://dx.doi.org/10.1016/j.amepre.2014.08.026.
5. Bleich SN, Wolfson JA, Jarlenski MP. Calorie changes in large chain restaurants: declines in new menu items but room for improvement. *Am J Prev Med*. **2016,** *50*, e1–e8. http://dx.doi.org/10.1016/j.amepre.2015.05.007.
6. Bruemmer B, Krieger J, Saelens BE, Chan N. Energy, saturated fat, and sodium were lower in entrées at chain restaurants at 18 months compared with 6 months following the implementation of mandatory menu labeling regulation in King County, Washington. *J Assoc Nutr Diet*. **2012,** *112*, 1169–1176. http://dx.doi.org/10.1016/j.jand.2012.04.019.
7. Dumanovsky T, Nonas CA, Huang CY, Silver LD, Bassett MT. What people buy from fast-food restaurants: caloric content and menu item selection, New York City 2007. *Obesity (Silver Spring)*. **2009,** *17*, 1369–1374. http://dxx.doi.org/10.1038/oby.2009.90.
8. Hearst MO, Harnack LJ, Bauer KW, Earnest AA, French SA, Michael Oakes J. Nutritional quality at eight U.S. fast-food chains: 14-year trends. *Am J Prev Med*. **2013,** *44*, 589–594. http://dx.doi.org/10.1016/j.amepre.2013.01.028.
9. Hill JL, Olive NC, Waters CN, Estabrooks PA, You W, Zoellner JM. Lack of healthy food options on children's menus of restaurants in the health-disparate Dan River region of Virginia and North Carolina, 2013. *Prev Chronic Dis*. **2015,** *12*, 140400. http://dx.doi.org/10.5888/pcd12.140400.
10. Jacobson MF, Havas S, McCarter R. Changes in sodium levels in processed and restaurant foods, 2005 to 2011. *JAMA Intern Med*. **2013,** *173*, 1285–1291. http://dx.doi.org/10.1001/jamainternmed.2013.6154.
11. Jarlenski MP, Wolfson JA, Bleich SN. Macronutrient composition of menu offerings in fast food restaurants in the U.S. *Am J Prev Med*. **2016,** *51*, e91–e97. http://dx.doi.org/10.1016/j.amepre.2016.03.023.
12. Kirkpatrick SI, Reedy J, Kahle LL, Harris JL, Ohri-Vachaspati P, Krebs-Smith SM. Fast-food menu offerings vary in diet quality, but are consistently poor. *Public Health Nutr*. **2014,** *17*, 924–931. http://dx.doi.org/10.1017/S1368980012005563.
13. Krukowski RA, Eddings K, West DS. The children's menu assessment: development, evaluation, and relevance of a tool for evaluating children's menus. *J Am Diet Assoc*. **2011,** *111*, 884–888. http://dx.doi.org/10.1016/j.jada.2011.03.018.
14. Moran AJ, Block JP, Goshev SG, Bleich SN, Roberto CA. Trends in nutrient content of children's menu items in U.S. chain restaurants. *Am J Prev Med*. **2017,** *52*, 284–291. http://dx.doi.org/10.1016/j.amepre.2016.11.007.

15. O'Donnell SI. Nutrient quality of fast food kids meals. *Am J Clin Nutr*. **2008,** *88*, 1388–1395. http://ajcn.nutrition.org/content/88/5/1388.long.
16. Rudelt A, French S, Harnack L. Fourteen-year trends in sodium content of menu offerings at eight leading fast-food restaurants in the USA. *Public Health Nutr*. **2014,** *17*, 1682–1688. http://dx.doi.org/10.1017/S136898001300236X.
17. Schoffman DE, Davidson CR, Hales SB, Crimarco AE, Dahl AA, Turner-McGrievy GM. The fast-casual conundrum: Fast-casual restaurant entrees are higher in calories than fast food. *J Acad Nutr Diet*. **2016,** *116*, 1606–1612. http://dx.doi.org/10.1016/j.jand.2016.03.020.
18. Serrano EL, Jedda VB. Comparison of fast-food and non-fast-food children's menu items. *J Nutr Educ Behav*. **2009,** *41*, 132–137. http://dx.doi:10.1016/j.jneb.2008.02.005.
19. Sliwa S, Anzman-Frasca S, Lynskey V, Washburn K, Economos C. Assessing the availability of healthier children's meals at leading quick-serve and full-service restaurants. *J Nutr Educ Behav*. **2016,** *48*, 242–249. http://dx.doi.org/10.1016/j.jneb.2016.01.004.
20. Urban LE, McCrory MA, Dallal GE, Das SK, Saltzman E, Weber JL, Roberts SB. Accuracy of stated energy contents of restaurant foods. *J Am Diet Assoc*. **2011,** *306*, 287–293. http://dx.doi.org/10.1001/jama.2011.993.
21. Urban LE, Lichtenstein AH, Gary CE et al. The energy content of restaurant foods without stated calorie information. *JAMA Intern Med*. **2013,** *173*, 1292–1299. http://dx.doi.org/10.1001/jamainternmed.2013.6163.
22. Urban LE, Roberts SB, Fierstein JL, Gary CE, Lichtenstein AH. Sodium, saturated fat, and trans fat content per 1,000 kilocalories: Temporal trends in fast-food restaurants, United States, 2000–2013. *Prev Chronic Dis*. **2014,** *11*, 140335. http://dx.doi.org/10.5888/pcd11.140335.
23. Urban LE, Weber JL, Heyman MB et al. Energy contents of frequently ordered restaurant meals and comparison with human energy requirements and US Department of Agriculture database information: a multisite randomized study. *J Acad Nutr Diet*. **2016,** *116*, 590–598.e6. http://dx.doi.org/10.1016/j.jand.2015.11.009.
24. Wu HW, Sturm R. What's on the menu? A review of the energy and nutritional content of US chain restaurant menus. *Public Health Nutr*. **2012,** *16*, 87–96. http://dx.doi.org/10.1017/S136898001200122X.
25. Wu HW, Sturm R. Changes in the energy and sodium content of main entrées in US chain restaurants from 2010 to 2011. *J Acad Nutr Diet*. **2014,** *114*, 209–219. http://dx.doi.org/10.1016/j.jand.2013.07.035.
26. Cohen DA, Lesser LI, Wright C, Story M, Economos C. Kid's menu portion sizes: how much should children be served? *Nutrition Today*. **2016,** *51*, 273–280.
27. Crixell SH, Friedman B, Fisher DT, Biediger-Friedman L. Improving children's menus in community restaurants: best food for families, infants, and toddlers (Best Food FITS) intervention, South Central Texas, 2010-2014. *Prev Chronic Dis*. **2014,** *11*, E223. http://dx.doi.org/10.5888/pcd11.140361.
28. Escaron AL, Martinez-Donate AP, Riggall AJ, Meinen A, Hall B, Javier Nieto F, Nitzke S. Developing and implementing "Waupaca Eating Smart": a restaurant and supermarket intervention to promote healthy eating through changes in the food environment. H*ealth Promot Pract*. **2016,** *17*, 265–277. http://dx.doi.org/10.1177/1524839915612742.

29. Gase L, Dunning L, Kuo T, Simon P, Fielding JE. Restaurant owners' perspectives on a voluntary program to recognize restaurants for offering reduced-size portions, Los Angeles County, 2012. *Prev Chronic Dis*. **2014,** *11*, E44. http://dx.doi.org/10.5888/pcd11.130310.
30. Gase LN, Kaur M, Dunning L, Montes C, Kuo T. What menu changes do restaurants make after joining a voluntary restaurant recognition program? *Appetite*. **2015,** *89*, 131–135. http://dx.doi.org/10.1016/j.appet.2015.01.026.
31. Wansink B, Hanks AS. Calorie reductions and within-meal calorie compensation in children's meal combos. *Obesity (Silver Spring)*. **2014,** *22*, 630–632. http://dx.doi.org/10.1002/oby.20668.
32. Anzman-Frasca S, Mueller MP, Sliwa S et al. Changes in children's meal orders following healthy menu modifications at a regional US restaurant chain. *Obesity (Silver Spring)*. **2015,** *23*, 1055–1062. http://dx.doi.org/10.1002/oby.21061.
33. Anzman-Frasca S, Mueller MP, Lynskey VM, Harelick L, Economos CD. Orders of healthier children's items remain high more than two years after menu changes at a regional restaurant chain. *Health Aff (Millwood)*. **2015,** *34*, 1885–1892. http://dx.doi.org/10.1377/hlthaff.2015.065.
34. Krukowski RA, West D. No financial disincentive for choosing more healthful entrées on children's menus in full-service restaurants. *Prev Chronic Dis*. **2013,** *10*, E94. http://dx.doi.org/10.5888/pcd.120266.
35. Peters JC, Beck J, Lande J, Pan Z, Cardel M, Ayoob K, Hill J. Using healthy defaults in Walt Disney World restaurants to improve nutritional choices. *J Assoc Consumer Res*. **2016,** *1*, 92–103. http://www.journals.uchicago.edu/doi/pdfplus/10.1086/684364.
36. Bernhardt AM, Wilking C, Adachi-Mejia AM, Bergamini E, Marijnissen J, Sargent JD. How television fast food marketing aimed at children compares with adult advertisements. *PLoS One*. **2013,** *8*, e72479. http://dx.doi.org/10.1371/journal.pone.0072479.
37. Bernhardt AM, Wilking C, Gottlieb M, Emond J, Sargent JD. Children's reaction to depictions of healthy foods in fast-food television advertisements. *JAMA Pediatrics*. **2014,** *168*, 422–426. http://dx.doi.org/10.1001/jamapediatrics.2014.140.
38. Bernhardt AM, Wilking C, Gilbert-Diamond D, Emond JA, Sargent JD. Children's recall of fast food television advertising-testing the adequacy of food marketing regulation. *PLoS One*. **2015,** *10*, e0119300. http://dx.doi.org/10.1371/journal.pone.0119300.
39. Elbel B, Mijanovich T, Cantor J, Bragg MA. New York City "Healthy Happy Meals" Bill: potential impact on fast food purchases. *Am J Prev Med*. **2015,** *49*, e45–e46. http://dx.doi.org/10.1016/j.amepre.2015.05.030.
40. Emond JA, Bernhardt AM, Gilbert-Diamond D, Li Z, Sargent JD. Commercial television exposure, fast food toy collecting, and family visits to fast food restaurants among families living in rural communities. *J Pediatrics*. **2016,** *168*, 158–163. http://dx.doi.org/10.1016/j.jpeds.2015.09.063.
41. Kraak VI, Story M. An accountability evaluation for industry's responsible use of brand mascots and licensed media characters to market a healthy diet to American children. *Obes Rev*. **2015,** *16*, 433–453. http://dx.doi.org/10.1111/obr.12279.
42. Ohri-Vachaspati P, Isgor Z, Rimkus L, Powell LM, Barker DC, Chaloupka FJ. Child-directed marketing inside and on the exterior of fast food restaurants. *Am J Prev Med*. **2015,** *48*, 22–30. http://dx.doi.org/10.1016/j.amepre.2014.08.011.

43. Otten JJ, Hekler EB, Krukowski RA et al. Food marketing to children through toys response of restaurants to the first U.S. toy ordinance. *Am J Prev Med*. **2012,** *42*, 56–60. http://dx.doi.org/10.1016/j.amepre.2011.08.020.
44. Otten JJ, Saelens BE, Kapphahn KI, Hekler EB, Buman MP, Goldstein BA, et al. (2014) Impact of San Francisco's toy ordinance on restaurants and children's food purchases, 2011–2012. *Prev Chronic Dis*. **2014,** *11*, 140026. http://dx.doi.org/10.5888/pcd11.140026.
45. Powell LM, Szczpka G, Chaloupka FJ. Trends in exposure to television food advertisements among children and adolescents in the United States. *Arch Pediatr Adolesc Med*. **2010,** *164*, 878–879. http://dx.doi.org/10.1001/archpediatrics.2010.139.
46. Anzman-Frasca S, Dawes F, Sliwa S et al. Healthier side dishes at restaurants: an analysis of children's perspectives, menu content, and energy impacts. *Int J Behav Nutr Phys Act*. **2014,** *11*, 81. http://dx.doi.org/10.1186/1479-5868-11-81.
47. Gase LN, Kaur M, Dunning L, Montes C, Kuo T. What menu changes do restaurants make after joining a voluntary restaurant recognition program? *Appetite*. **2015,** *89*, 131–135. http://dx.doi.org/10.1016/j.appet.2015.01.026.
48. Henry HK. Borzekowski DL. Well, that's what came with it. A qualitative study of U.S. mothers' perceptions of healthier default options for children's meals at fast-food restaurants. *Appetite*. **2015,** *87*, 108–115. http://dx.doi.org/10.1016/j.appet.2014.12.201.
49. Bleich SN, Wolfson JA, Jarlenski MP, Block JP. Restaurants with calories displayed on menus had lower calorie counts compared to restaurants without such labels. *Health Aff (Millwood)*. **2015,** *34*, 1877–1884. http://dx.doi.org/10.1377/hlthaff.2015.0512.

Supplemental Table 2 Gray literature used to evaluate the U.S. restaurant industry's progress to create healthy food environments for American customers, 1 January 2006–30 January 2017

**Lead Author, Year**	**Data collection period (years)**<br>**Study design**	**Data sources**<br>**Assessment tools or Report**	**Restaurants sampled**<br>**Study location**	**Results and outcomes measured**
**1. PLACE** ***(Improve the ambience or atmosphere to promote healthy restaurant food and beverage offerings)*** (n=0)				
No reports identified				
**2. PROFILE** *(Change the nutrient composition to provide food and beverage offerings that promote a healthy diet)* (n=7)				
Batada 2013 [1]	Oct–Nov 2012<br><br>Descriptive	Restaurant website/headquarters<br><br>USDA Nutrition Analysis Tool<br><br>National Alliance for Nutrition and Activity (NANA) expert nutrition standards based on the Dietary Guidelines for Americans 2010<br><br>*Kids LiveWell* Program nutrition standards 2011	34 quick-service restaurants (QSRs), fast casual restaurants (FCRs) and full-service restaurant (FSR) chains including:<br>Buffalo Wild Wings<br>Carl's Jr.<br>Chipotle<br>Dairy Queen<br>Hardee's<br>McDonald's<br>Panda Express<br>Perkins<br>Popeye's<br>Whataburger<br>Ruby Tuesday<br>Chili's<br>Red Robin<br>Quizno's<br>T.G.I Fridays<br>KFC<br>Panera<br>P.F. Changs<br>Taco Bell	Only 3% of 3,494 children's meal combinations at 34 restaurant chains met expert-defined nutrition standards, and only 9% met the nutrition standards of the National Restaurant Association's (NRA's) *Kids LiveWell* Program.

			Olive Garden<br>Outback Steakhouse<br>Jack in the Box<br>Applebee's<br>Sonic<br>Wendy's<br>Longhorn Steakhouse<br>Bob Evans<br>Denny's<br>Chick-fil-A<br>Arby's<br>Burger King<br>Red Lobster<br>IHOP<br>Subway	
Cardello 2013 [2]	2006–2011<br><br>Descriptive<br><br>Longitudinal	Trinity Capital<br>Corporate Annual News Reports<br>Nation's Restaurant News<br>NPD/Crest	21 QSR, FCR and FSR chains including:<br>Applebee's<br>Arby's<br>Burger King<br>Carrabba's<br>Cracker Barrel<br>Chick-fil-A<br>Chili's<br>Denny's<br>IHOP<br>Kentucky Fried Chicken (KFC)<br>Longhorn Steakhouse<br>McDonald's<br>Olive Garden<br>On the Border<br>Outback Steakhouse<br>Panera Bread<br>Red Lobster<br>Romano's Macaroni Grill<br>Sonic<br>Taco Bell<br>Wendy's	A total of 17 of 21 leading restaurant chains expanded the availability and sale of lower-calorie items (e.g., ≤ 50 kcal/serving for beverages, ≤ 150 kcal/serving for side dishes/appetizers/desserts, and ≤ 500 kcal/serving for meals) that were linked to increased customer traffic and a 10% increase in profitable sales growth.

Center for Science in the Public Interest, 2014 [3]	2009–2013<br><br>Descriptive	Restaurant websites and phone calls<br><br>DGA 2010	17 QSR and FSR chains including:<br>Arby's<br>Burger King<br>Chick-fil-A<br>Chili's<br>Dairy Queen<br>Denny's<br>Jack in the Box<br>Kentucky Fried Chicken<br>McDonald's<br>Olive Garden<br>Panera Bread<br>Pizza Hut<br>Red Lobster<br>Sonic<br>Subway<br>Taco Bell<br>Wendy's	Nine out of 17 restaurants revealed a trend toward reducing the sodium content of foods, while the remaining 8 restaurants had increased the sodium in the offerings over 4 years.<br>The average sodium content of the 55 children's meals examined decreased by 2.6% over the four-year study period.<br>Meals that contained more than a day's worth of sodium (1,200 mg) recommended for children ages 4-8 years (1,200 mg) declined by 17%.<br>Subway reduced the sodium content in children's meals by 29% and had the lowest average sodium content for children's meals (600 mg/meal).<br>About 44% of children's meals sampled contained more than 1,200 mg of sodium in 2013.
Center for Science in the Public Interest, 2014 [4]	2012, 2014	MenuStat	25 QSR, FCR and FSR chains including:<br>McDonald's<br>Subway<br>Starbucks<br>Burger King<br>Wendy's<br>Taco Bell<br>Dunkin' Donuts<br>Pizza Hut<br>KFC<br>Applebee's<br>Chick-Fil-A<br>Sonic<br>Olive Garden<br>Chili's<br>Domino's<br>Panera Bread<br>Jack in the Box<br>Arby's	Between 2012 and 2014, the average sodium content across all menu items declined only by 1% from 1267 mg to 1256 mg. Across all 163 children's items sampled, the sodium content decreased by 8% from 619 mg to 597 mg.<br>The sodium levels increased by 2% for items that were on the menus for both 2012 and 2014, from 585 mg to 597 mg. Children's entrees, appetizers, and side dishes had 2-8% increases in sodium content. These results suggest that restaurants are not reformulating existing options, but rather adding new lower sodium options. Applebee's, IHOP, and Dairy Queen had the highest sodium

			Dairy Queen<br>Red Lobster<br>IHOP<br>Denny's<br>Outback Steakhouse<br>Chipotle<br>Papa John's	in children's meals of the 25 restaurants sampled.
Harris et al., 2010 [5]	2009<br><br>Descriptive	The Nielsen Company, comScore Inc., Arbitron Inc., NPD Group<br><br>National School Lunch Program established by the IOM Committee on School Meals<br><br>Nutrient Profiling Index (NPI) Score	12 QSR chains including:<br>McDonald's<br>Subway<br>Burger King<br>Starbucks<br>Wendy's<br>Taco Bell<br>Pizza Hut<br>Dunkin' Doughnuts<br>KFC<br>Sonic<br>Domino's<br>Dairy Queen	Only 12 out of 3,039 children's meal combinations at 12 leading QSR chains met the Institute of Medicine's (IOM's) School Meals nutrition criteria for preschoolers (≤ 410 calories and 544 mg sodium/meal); 15 meals met the IOM's nutrition criteria for older children (≤ 650 calories and 636 mg sodium/meal); meals purchased by adolescents provided an average of 800-1,100 calories/meal representing half of their recommended daily calories; and meals sold to young people rarely offered healthy side dishes as the default choice.
Harris et al., 2013 [6]	2012–2013<br>Descriptive	NPI Score based on the IOM calorie and sodium limits<br><br>Kids LiveWell nutrition standards<br><br>Children's Food and Beverage Advertising Initiative uniform standards<br><br>Federal Interagency Working Group (IWG) guidelines	12 QSR chains including:<br>McDonald's<br>Subway<br>Wendy's<br>Burger King<br>Taco Bell<br>Chick-fil-A<br>KFC<br>Panera Bread<br>Sonic<br>Jack in the Box<br>Arby's<br>Dairy Queen	Less than one percent of children's meals met four different nutrition standards.

Wootan et al., 2008 [7]	2008<br><br>Descriptive	Corporate Headquarters National Alliance for Nutrition and Activity's Model Local School Wellness Policies on Physical Activity and Nutrition	25 QSR and FCR chains including:<br>Buffalo Wild Wings<br>Carl's Jr.<br>Chipotle<br>Dairy Queen<br>Hardee's<br>McDonald's<br>Panda Express<br>Perkins<br>Popeye's<br>Whataburger<br>Ruby Tuesday<br>Chili's<br>Red Robin<br>Quizno's<br>T.G.I Fridays<br>KFC<br>Panera<br>P.F. Changs<br>Taco Bell<br>Olive Garden<br>Outback Steakhouse<br>Jack in the Box<br>Applebee's<br>Sonic<br>Wendy's<br>Longhorn Steakhouse<br>Bob Evans<br>Denny's<br>Chick-fil-A<br>Arby's<br>Burger King<br>Red Lobster<br>IHOP<br>Subway	Six of 25 QSR and FCR chains did not offer children's menus, and 93 percent of the children's meals analyzed provided excessive calories, saturated fat and sodium.

**3. PORTION** ***(Reduce the size of children's meals and components to meet recommended nutrition criteria)*** (n=0)				
No resources identified				
**4. PRICING** ***(Increase customers' purchases and the restaurants' revenue for healthy restaurant food and beverage offerings)*** (n=0)				
No resources identified				
**5. PROMOTION** ***(Use responsible marketing practices to promote healthy food and beverage offerings for children and adolescents)*** (n=8)				
Council of Better Business Bureaus, 2014 [8]	Jan 2014 Report	CFBAI's uniform nutrition standards	Two QSR chains including:<br>Burger King<br>McDonald's	The CFBAI adopted new uniform criteria for calories, nutrients to limit, and nutrition components to encourage effective December 31, 2013.
Council of Better Business Bureaus, 2016 [9]	2016 Report	Not relevant	Two QSR chains including:<br>Burger King<br>McDonald's	McDonald's and Burger King are the only two restaurant chains that participate in the CFBAI.
Frazer and Harris, 2016 [10]	June 2016 Report	The Nielsen Company data to assess changes in TV advertising exposure between 2014 and 2015	QSR chains and other restaurants advertising trends	From 2014-2015, individuals in all age groups continued to see more ads for QSRs than any other food category, representing 29% of food-related TV ads viewed by children and more than 33% of food-related TV ads viewed by adolescents.
Harris et al., 2010 [5]	2009<br><br>Descriptive	The Nielsen Company, comScore Inc., Arbitron Inc., NPD Group<br><br>National School Lunch Program established by the IOM Committee on School Meals<br><br>Nutrient Profiling Index(NPI) Score	12 QSR chains including:<br>McDonald's<br>Subway<br>Burger King<br>Starbucks<br>Wendy's<br>Taco Bell<br>Pizza Hut<br>Dunkin' Doughnuts<br>KFC<br>Sonic<br>Domino's<br>Dairy Queen	On average, preschoolers viewed 2.8 fast food ads every day, children viewed 3.5 ads, and teens viewed 4.7 ads per day. McDonald's, Burger King, and Yum Brands! constituted 60% of fast food ads seen by children, and 50% of those seen by teens. Between 2007 and 2009, McDonald do 26% and Burger King increase advertising to children ages 6-11 years by 10%.

Harris et al., 2013 [6]	2012–2013<br><br>Descriptive	NPI score<br><br>Calorie and sodium levels recommended by several sources:<br>IOM<br>NRA's Kids *LiveWell* program<br>CFBAI uniform nutrition standards<br>Interagency Working Group guidelines	18 QSR and FCR chains including:<br>McDonald's<br>Subway<br>Starbucks<br>Wendy's<br>Burger King<br>Taco Bell<br>Dunkin' Donuts<br>Pizza Hut<br>Chik-fil-A<br>KFC<br>Panera Bread<br>Sonic<br>Domino's<br>Jack in the Box<br>Arby's<br>Dairy Queen<br>Little Caesars<br>Cici's Pizza	Only one third of TV ads promoted healthier kid's meals. The average preschooler viewed 2.8 ads per day in 2012, children (6-11) viewed 3.2 ads per day, and teens (12-17) viewed 4.8 ads per day.
Harris et al., 2015 [11]	2012–2014<br><br>Descriptive	Nielsen Data	13 QSR and FSR chains including:<br>McDonald's<br>Taco Bell<br>KFC<br>Pizza Hut<br>Wendy's<br>Burger King<br>Arby's<br>Carl's Jr.<br>Hardees<br>Applebee's<br>IHOP<br>Sonic<br>Olive Garden	McDonald's was one of the largest advertising spenders in African American and Hispanic-focused media. Fast food in general spent the most in the food, beverage, and restaurant sector on minority advertising, with $244 million spent on Spanish-language TV and $61 million in African American-targeted TV.
Leibowitz et al., 2012 [12]	2006-2009<br><br>Descriptive	Expenditures and data submitted to the Federal Trade Commission of 48 U.S. food, beverage, restaurant and entertainment companies	QSRs were not identified	Quick-service restaurant (QSR) foods, carbonated beverages, and breakfast cereals accounted for $1.29 billion of all youth-directed expenditures. The reporting QSRs spent $714 million on youth marketing in 2009 (down from $733

				million in 2006), with $583 million directed to children and $183 million directed to teens (including overlap of the two age groups).<br><br>The drop in child-directed QSR expenditures was due primarily to reduced premium expenditures, and attributed to restaurants selling fewer kids' meals with toys, and the costs of these toys was less in 2009 versus 2006. The increase in teen-directed expenditures for QSR stemmed primarily from greater television expenditures as well as modest increases in radio and new media advertising.<br><br>Five QSR companies reported data for both 2006 and 2009. Four additional companies were added in 2009. For the five QSR companies reporting in both years, there was some improvement from 2006 to 2009 for child-directed TV advertising where products averaged 79 fewer calories, 57 mg less sodium, 6 g less sugar, and 0.5 g less saturated fat. In teen-directed TV advertising, improvements were more modest; products averaged 43 fewer calories, 14 mg less sodium, and 1 g less sugar. The positive nutritional changes were due to marketing of newer and more nutritious menu items.<br><br>Celebrity endorsement, which represent a non-measured media activity, was among several

				activities that accounted for about $315 million or 17.6% of all reported youth-directed marketing expenditures in 2009.<br><br>The six QSRs analyzed reported small expenditures for youth-directed athletic sponsorships ($1.6 million) and celebrity endorsement fees ($2.5 million). Half of all child-directed marketing dollars ($530.7 million) in 2009 involved cross-promotions. QSRs ($428.2 million) accounted for 81% of that amount.<br><br>The 48 companies spent a total of $99.3 million on youth-directed promotions featuring celebrity endorsers, representing 5.6% of all youth-directed spending, a substantial increase from the $26.8 million spent in 2006. In 2009, QSRs spent about $1 million on celebrity marketing to children and $7 million on teens to promote restaurant foods.<br><br>The FTC noted that restaurant franchisees, independent distributors, and local bottlers for CFBAI member companies did not always adhere to the member companies' pledge commitments.
Wescott et al., 2015 [13]	2014–2015	Nielsen Data	One QSR chain:<br>McDonald's Corporation	In 2013, McDonald's partnered with the Alliance for a Healthier Generation and agreed to make five commitments including:<br>1) feature only milk, water, and juice as the beverage in Happy

				Meals on child-directed advertisements 2) offer a fruit or vegetable side as a substitute for fries in value meals 3) use Happy Meal and other packaging innovations to generate excitement for healthy options 4) dedicate one portion of the Happy Meal container to communicate a health-promoting message; and<br>5) 100% of advertising to children must include a fun nutrition or well-being message.<br><br>Results showed that McDonald's Corporation met commitments 1, 3, 4 and 5 in 2014 and made progress on commitment 2 in 2015.
**6. Healthy Default PICKS** ***(Use environmental cues to socially normalize healthy default food and beverage choices)*** (n=4)				
Batada et al., 2013 [1]	Oct–Nov 2012<br><br>Descriptive	Restaurant website/headquarters<br><br>USDA Nutrition Analysis Tool	34 QSR, FCR and FSR chains including:<br>Buffalo Wild Wings<br>Carl's Jr<br>Chipotle<br>Dairy Queen<br>Hardee's<br>McDonald's<br>Panda Express<br>Perkins<br>Popeye's<br>Whataburger<br>Ruby Tuesday<br>Chili's<br>Red Robin<br>Quizno's<br>T.G.I Fridays<br>KFC<br>Panera<br>P.F. Changs<br>Taco Bell	More than three quarters (78%) of restaurant chains offered soft drinks as a children's beverage option; 40% offered desserts specifically for children; 73% of restaurant chains offered fried potatoes as a side; 53% had a non-potato vegetable side available; and 68% offered a fruit side.<br><br>All of the children's meals sold by Subway included apple slices and a choice of apple juice, low-fat milk, or bottled water, and met the expert and Kids *LiveWell* nutrition standards.

			Olive Garden<br>Outback Steakhouse<br>Jack in the Box<br>Applebee's<br>Sonic<br>Wendy's<br>Longhorn Steakhouse<br>Bob Evans<br>Denny's<br>Chick-fil-A<br>Arby's<br>Burger King<br>Red Lobster<br>IHOP<br>Subway	
Clinton Global Initiative [14]	2013	Clinton Global Initiative Commitments to Action Correspondence with McDonald's Corporation	One restaurant chain:<br>McDonald's Corporation	McDonald's agreed to make five commitments described below that were effective 2015 and will be fully implemented by 2020:<br><br>1. feature only milk, water, and juice as the beverage in Happy Meals on child-directed advertisements;<br>2. offer a fruit or vegetable side as a substitute for fries in value meals;<br>3. use Happy Meal and other packaging innovations to generate excitement for healthy options;<br>4. dedicate one portion of the Happy Meal container to communicate a health promoting message; and<br>5. 100% of advertising to children must include a fun nutrition or well-being message. McDonalds met commitments 1,3,4, and 5 in 2014 and made progress on commitment #2 in 2015.
National Fruit and Vegetable Alliance, 2010 [15]	2006-2010 (period of interest) although	MenuTrends data	Specific restaurants were not identified	The restaurant sector was awarded a grade for its performance on a scale of A to F for providing menu

	data collection began in 2005			offerings that contained fruits or vegetables. The grades included: vegetable items (C), fruit items (D), and for the percentage of fruits and vegetables consumed at meals by customers (F).<br><br>In 2009, the number of menu items that included vegetables had slightly increased from 2005 to 44.8%, and the number of menu items that included fruit had increased to 8.8%. Fruit was found on menus five times less than vegetables. The proportion of fruits and vegetables consumed in restaurants had decreased 0.6% to 11% in 2009.
National Fruit and Vegetable Alliance, 2015 [16]	2010–2015<br><br>Descriptive	MenuTrends data	Specific restaurants were not identified	Restaurants increased fruit and vegetable menu availability by 28% from 2012 to 2014 but were awarded a B- on summary of progress from 2010 through 2015. In 2010, vegetables were offered 5 times more often then fruit, by 2014, there was a slight decline to 4.3 times more often. Though reported ease in finding fruits and vegetables on restaurant menus has increased, the proportion of fruits and vegetables consumed at restaurants, 4% and 16%, respectively, has remained constant from 2009-2014.
**7. PRIMING or PROMPTING** *(Provide information to customers to promote healthy food and beverage choices at restaurants)* (n=2)				

Government Accountability Office, 2010 [17]	2010	"Nutrition labeling of standard menu items at chain restaurants" of the Patient Protection and Affordable Care Act of 2010, Public Law 111–148 (H.R. 3590).	Not applicable	This law outlined the mandatory restaurant menu labeling for chain restaurants with 20 or more locations across the United States. This law required chain restaurants to modify their menu offerings and marketing practices by 5 May 2017 to meet the FDA requirement to provide calorie information and specific language regarding daily calorie intake on menus and menu boards, and additional nutrition information to customers upon request.
New York City Department of Health and Mental Hygiene, 2015 [18]	2015	National Salt Reduction Initiative	New York City restaurants with 15 or more locations	Beginning December 1, 2015, Restaurants with 15 or more locations in New York City were required to post warning labels and a salt symbol next to menu items that exceeded a full day's worth of recommended sodium (2,300 mg/day).
**8. PROXIMITY** *(Make healthy choices at eye level and physically closer to customers at point of choice or purchase)* (n=0)				
No resources identified				

Abbreviations

Dietary Guidelines for Americans (DGA), fast-casual restaurants (FCR), Food and Drug Administration (FDA), full-service restaurants (FSR), gram (g), kilocalories (kcal), limited-service restaurants (LSRs), National Restaurant Association (NRA), quick-service restaurants (QSR), television (TV) and U.S. Department of Agriculture (USDA)

References

1. Batada A. *Kids' Meals: Obesity on the Menu*. Washington, DC: Center for Science in the Public Interest, March 2013. Available online: http://cspinet.org/new/pdf/cspi-kids-meals-2013.pdf (accessed 15 May 2017).
2. Cardello H. *Lower-Calorie Foods: It's Just Good Business*. Obesity Solutions Initiative. Washington, DC: The Hudson Institute, February 2013. Available online: http://www.rwjf.org/content/dam/farm/reports/reports/2013/rwjf404136 (accessed 15 May 2017).
3. Center for Science in the Public Interest. *Stalling on Salt. Restaurant Meals Still Loaded with Sodium*. June 2014. Available online: http://cspinet.org/new/pdf/stalling_on_salt.pdf (accessed 15 May 2017).
4. Center for Science in the Public Interest. *Restaurants Can't Shake the Salt*. 2014. Available online: http://cspinet.org/images/salt-report-draft.pdf (accessed 15 May 2017).
5. Harris JL, Schwartz MB, Brownell KD, Sarda V, Ustjanauskas A, Javadizadeh J, Weinberg M, Munsell C, Speers S, Bukofzer E, Cheyne A, Gonzalez P, Reshetnyak J, Agnew H, Ohri-Vachaspati P. *Fast Food FACTS: Evaluating Fast Food Nutrition and Marketing to Youth*. Rudd Center for Food Policy and Obesity, 2010. Available online: http://www.rwjf.org/content/dam/farm/reports/reports/2010/rwjf68794 (accessed 15 May 2017).
6. Harris JL, Schwartz MB, Munsell CR, Dembek C, Liu S, LoDolce M, Heard A, Fleming-Milici F. *Fast Food Facts 2013: Measuring Progress in the Nutritional Quality and Marketing of Fast Food to Children and Teens*. Yale Rudd Center for Food Policy & Obesity, November 2013. Available online: http://www.rwjf.org/content/dam/farm/reports/reports/2013/rwjf408549 (accessed 15 May 2017).
7. Wootan M, Batada A, Marchlewicz E. *Kids' Meals: Obesity on the Menu*. Washington, DC: Center for Science in Public Interest, 2008. Available online: www.cspinet.org/kidsmeals (accessed 15 May 2017).
8. Council of Better Business Bureaus, Inc. (CBBB). Children's Food & Beverage Advertising Initiative. *Foods and Beverages that Meet the CFBAI Category-Specific Uniform Nutrition Criteria that May Be in Child-Directed Advertising*. 2014. Available online: http://www.bbb.org/globalassets/local-bbbs/council-113/media/cfbai/cfbai-product-list-january-2014.pdf (accessed 15 May 2017).
9. Council of Better Business Bureaus, Inc. (CBBB). *The National Partner Program. Welcome to the Children's Food and Beverage Advertising Initiative*. 2016. Available online: http://www.bbb.org/council/the-national-partner-program/national-advertising-review-services/childrens-food-and-beverage-advertising-initiative/ (accessed 15 May 2017).
10. Frazer III WC, Harris JL. *Trends in television food advertising to young people: 2015 update*. University of Connecticut Rudd Center for Food Policy & Obesity. June 2016. Available online: http://www.uconnruddcenter.org/files/Pdfs/RuddReport_TVFoodAdvertising_6-2016.pdf (accessed 15 May 2017).
11. Harris JL, Shehan C, Gross R (Rudd Center); Kumanyika S, Lassiter V (AACORN); Ramirez AG, Gallion K, (Salud America!). *Food advertising targeted to Hispanic and Black youth: Contributing to health disparities*. University of Connecticut: Rudd Center for Food Policy & Obesity, 2015. Available online: http://www.uconnruddcenter.org/files/Pdfs/272-7%20%20Rudd_Targeted%20Marketing%20Report_Release_081115%5B1%5D.pdf (accessed 15 May 2017).

12. Leibovitz J, Rosch JT, Ramirez E, Brill J, Ohlhausen M. *A review of food marketing to children and adolescents: follow-up report.* Washington, DC: U.S. Federal Trade Commission, 2012. Available online: http://www.ftc.gov/os/2012/12/121221foodmarketingreport.pdf (accessed 15 May 2017).
13. Wescott RF, Fitzpatrick B, Phillips E. *2014 Progress Report.* McDonald's-Alliance for a Healthier Generation Partnership: Clinton Global Initiative Commitment to Action. Washington, DC: Keybridge. 15 July 2015. Available online: https://www.healthiergeneration.org/_asset/qqj3bi/2014-Progress-Report_McDonalds-Alliance-Partnership-on-CGI-Commitment-t---.pdf (accessed 15 May 2017).
14. Clinton Global Initiative. ID 1620022. *2013 CGI Commitment to Action. Commitment by McDonald's Corporation.* September 2013. Available online: https://www.healthiergeneration.org/_asset/t5xpix/2013-AM_Health_McDonalds_Happier-Happy-Meals_1620022_Final_AB.pdf (accessed 15 May 2017).
15. National Fruit and Vegetable Alliance. *National Action Plan to Promote Health through Increased Fruit and Vegetable Consumption 2010 Report Card.* Produce for Better Health Foundation, 2010. Available online: http://www.nfva.org/pdfs/nfva/FINALNAP2010.pdf (accessed 15 May 2017).
16. National Fruit and Vegetable Alliance. *National Action Plan to Promote Health through Increased Fruit and Vegetable Consumption 2015 Report Card.* Produce for Better Health Foundation, 2015. Available online: http://www.nfva.org/national_action_plan.html (accessed 15 May 2017).
17. Section 4205 *"Nutrition labeling of standard menu items at chain restaurants" of the Patient Protection and Affordable Care Act of 2010, Public Law 111–148 (H.R. 3590).* 23 March 2010. Available online: http://www.gpo.gov/fdsys/pkg/PLAW-111publ148/pdf/PLAW-111publ148.pdf (accessed 15 May 2017).
18. New York City Department of Health and Mental Hygiene. *Sodium Initiatives*. 2015. Available online: http://www.nyc.gov/html/doh/html/diseases/salt.shtml (accessed 15 May 2017).

Supplemental Table 3 Media stories or news releases used to evaluate the U.S. restaurant industry's progress to create healthy food Environments for American customers, 1 January 2006–30 January 2017

**Arranged in chronological order*

**Source, Date**	**Title**	**Description**
Fast Casual, 17 July 2006 [1]	NRA joins Healthy Dining to promote healthful menu choices	The National Restaurant Association and the California-based Healthy Dining Program launched a Website to help Americans easily identify smart choices on restaurant menus. The Website was developed with partial funding from the Centers for Disease Control and Prevention and represents a collaborative effort by the restaurant industry to support public health. HealthyDiningFinder.com is a free resource that will enable customers to search for participating restaurants based on location, type of cuisine, price range, takeout availability and other criteria. Detailed nutrition information (calories, fat, saturated fat, cholesterol, sodium, protein, carbohydrates, fiber and fruit/vegetable servings) is provided for each menu item featured.
National Restaurant Association, 27 July 2009 [2]	Produce and foodservice leaders set goal to double produce use in 10 years.	Produce and foodservice industry leaders announced that they have set an ambitious goal to double use of fresh produce in the foodservice sector over the next 10 years, and have identified five strategies for doing so. The goal was set during an invitation-only "Executive Think Tank" discussion of senior foodservice and produce industry leaders. The five priority areas that participants identified to help achieve that goal of doubling produce use include: (1) re-imagine the restaurant experience, with produce having a

		stronger presence and telling its story from field to fork; (2) increase consumer confidence in fresh produce, including product safety, trust, and integrity; (3) demonstrate social responsibility, balancing the needs of people, the planet, and profitability; (4) foster closer collaboration among the industry sectors, including operators, distributors, and grower/shippers; and (5) foster closer collaboration with government and other stakeholders.
National Restaurant Association, 22 March 2010 [3]	National Restaurant Association says nutrition information provision is win for consumers and restaurants.	The National Restaurant Association issued a statement praising passage of a provision that will provide more nutrition information to consumers in chain restaurants across the country. The rule was a component of healthcare legislation that passed the U.S. House of Representatives Sunday and is expected to be signed into law by President Obama. The agreement creates a national, uniform standard for chain restaurants with 20 or more locations that would provide customers with a wide range of nutrition information—mirroring the information available on packaged foods—at the point of purchase. Caloric information would be highlighted on menus, menu boards, and drive-thru boards. “The passage of this provision is a win for consumers and restaurateurs,” said Dawn Sweeney, National Restaurant Association President and CEO. “We know the importance of providing consumers with the information they want and need, no matter in which part of the country they are dining. This legislation will replace a growing patchwork of varying state and local regulations with one consistent national standard that helps consumers make choices that are best for themselves and their families.”

White House Office of the First Lady, 13 September 2010 [4]	Remarks by the First Lady in address to the National Restaurant Association meeting.	First Lady Michelle Obama presented to the National Restaurant Association's annual meeting and acknowledged that while restaurants are offering more options and families are taking advantage of them, they are not always the healthiest choices. She asked the restaurant industry sector to provide children and families with healthier options.
Slossen, Reuters, 22 June 2011 [5]	Jack in the Box yanks toy from kids' meals.	The San-Diego-based company, Jack in the Box, announced that it would no longer offer toys with children's meals in order to promote healthier options for kids and parents. This decision received praise from public health advocacy organizations.
National Restaurant Association, 13 July 2011 [6]	Kids, parents praise NRA's Kids LiveWell program.	Nineteen leading restaurant companies representing more than 15,000 restaurants across the U.S. announced the launching of Kids LiveWell, a new National Restaurant Association kids' dining initiative. Launched with Healthy Dining, an organization with a nutrition-analysis company and consumer website, the Kids LiveWell Program shared restaurants that offer children's menu items that meet strict and healthy nutrition criteria. To qualify for Kids LiveWell, restaurants must offer at least one children's meal that includes an entrée, side and beverage for no more than 600 calories. Meals must consist of nutrient-rich foods, including two servings of fruit, vegetables, whole grain, lean protein and/or low-fat dairy; and also must limit sodium, fat and sugar. Participating restaurants must offer at least one other side dish that follows similar criteria. They also must provide nutrition information about the meal

		and promote the items so they are easily identifiable. The Kids LiveWell nutrition criteria are based on the 2010 USDA Dietary Guidelines, the Institutes of Medicine, government guidance for school nutrition and other health organizations.
Bryson, Chicago Tribune,<br>5 March 2012 [7]	McDonald's to kids: Eat fruit, drink milk, visit Arches. New ads that include nutritional, physical activity message to debut Friday.	McDonald's announced it would be beginning its new child directed advertising in 2012. All new child-directed advertising would meet the pledge McDonald's made to include a nutritional or physical activity message.
BlackPRWire (BPRW),<br>6 March 2012 [8]	McDonald's USA's new happy meal campaign to engage families in the benefits of active play, balanced eating.	McDonald's introduced a new campaign aimed at making nutrition and active lifestyles fun for children. The company stated that 100% of its national marketing efforts to children will include a health promoting message. The company also announced that its Happy Meals will offer default sides of apple slices and kid-sized fries.
National Restaurant Association,<br>26 February 2013 [9]	NRA honored for Kids *LiveWell* campaign.	In February 2016, the National Restaurant Association (NRA) was awarded an honorable mention by Public Relations Daily for the best public relations campaign for the issue/advocacy category.
Taco Bell, 23 July 2013 [10]	Taco Bell becomes first quick service restaurant to discontinue kid's meals and toys nationally.	Taco Bell announced in July 2013 that it will discontinue kid's toy premiums at its U.S. locations, being the first lead quick service chain to do so. Taco Bell CEO Greg Creed explained it was a move to improve Taco Bell and keep it relevant.

Subway, 23 January 2014 [11]	Subway Restaurants answers First Lady Michelle Obama's call to market healthier choices to kids.	Subway announced joining the Partnership for a Healthier America in a three-year commitment to promote healthier choices to children, including: fun campaigns to increase fruit and vegetable intake; establish and implement new marketing standards for its *Fresh Fit for Kids* meals; and provide healthy default choices in children's meals such as low-fat or non-fat milk or water as the default beverage and fruit as the default side dish.
Center for Science in the Public Interest, 30 January 2014 [12]	Restaurant chains urged to dump soda from kids' menus.	Over 100 national and local health organizations along with over 60 nutrition experts are urging restaurants to discontinue offering sodas among other sugar sweetened beverages from their children's menus. McDonald's, Subway, Chipotle, Arby's and Panera have excluded sodas as options for kids' meals.
O'Reilly, Business Insider, 15 May 2015 [13]	McDonald's slapped down for focusing its Happy Meal advertising on the toy and not the food.	The Children's Advertising Review Unit warned McDonald's Corporation that its Happy Meal advertisement was focused too heavily on the toy premium and not the meal. McDonald's removed the advertisement following the criticism.
Center for Science in the Public Interest, 27 May 2015 [14]	City of Davis, CA takes soda off kids' menus.	The city council in Davis, CA passed an ordinance banning soda as a default drink in kids' meals and instead allowing only milk or water.
McDonald's Corporation, 25 June 2015 [15]	McDonald's and Alliance for a Healthier Generation Announce progress on commitment to promote balanced food and beverage choices.	McDonald's partnered with the Alliance for a Healthier Generation and announced that they the company would work on a global

		commitment to increase customer's access to fruits and vegetables. McDonald's would also work to help families and children make healthful choices through 5 specific commitments to be fully completed by 2020.
Food and Drug Administration, 9 July 2015 [16]	FDA statement on extension of menu labeling compliance date.	The Food and Drug Administration, after reviewing comments and stakeholder input from food retailer establishments, announced it would extend the date for the menu labeling rule to December 1, 2016. This extension is a year past the original ruling to comply with menu labeling regulations.
Center for Science in the Public Interest, 30 November 2015 [17]	Applebee's to remove soda from kids menus.	Applebee's became the first family-dining restaurant chain to eliminate soda from its children's menus.
Aubrey and Godoy, National Public Radio, 1 December 2015 [18]	High-sodium warnings hit New York City menus.	A new sodium-warning requirement will take effect in New York City restaurants on December 1, 2015. Diners who eat at chain restaurants will now see warnings on menus next to items that contain high levels of salt. The New York City Health Department says chain restaurants with 15 or more locations must display a salt shaker icon next to menu items or combo meals that contain 2,300 milligrams of sodium or more. New York City is the first city in the U.S. to require a sodium warning. The rule applies to restaurants with 15 locations anywhere in the U.S.—not just in NYC—that will have until March 1, 2016 to comply or will be fined after that date.

National Restaurant Association, 4 December 2015 [19]	NRA sues NYC over sodium-labeling mandate.	Restaurant Association has filed suit against New York City's Board of Health over its decision to require chain restaurants with at least 15 stores to post sodium labeling next to menu items or combination meals exceeding 2,300 milligrams of sodium. The suit was filed in the Supreme Court of the State of New York. The city's sodium-labeling mandate took effect Dec. 1, and fines for noncompliance are expected to begin March 1, 2016. The NRA said it filed the lawsuit because the board of health lacks authority to enact such a mandate. Under New York State law, only the City Council has the right to impose mandates like this. The NRA also said the regulation is "arbitrary and capricious" and "filled with irrational exclusions and nonsensical loopholes." The regulation also undermines the FDA's federal menu-labeling law set to take effect in December 2016. That law will require chain restaurants to provide nutrition information on request, including sodium content.
Madhani, USA Today, 19 January 2016 [20]	McDonald's nears sale of 2 billion servings of fruit, yogurt.	McDonald's announced that it is approaching $2 billion dollars in sales of fruit and low-fat yogurt side dishes in its Happy Meals that the company began providing as default choices in 2011.

Gartland, New York Post, 27 January 2016 [21]	Lawmakers attempt at 'healthy' Happy Meals falls short.	The Deputy Health Commissioner of New York City told a City Council Health Committee hearing that it would be too tough to enforce proposed nutritional restrictions on the calorie-laden kids' meals. A council bill would require any kids' meal with an accompanying toy to be limited to no more than 500 calories and 600 milligrams of sodium, with no more than 35 percent of the calories from fat and no more than 10 percent from saturated fat or added sugar. The Commission said that restaurants will not be able to comply with, and the department will not be able to enforce, the bill's requirements. McDonald's Corporation had spent more than $528,000 to lobby council members to block the bill since 2011.
Center for Science in the Public Interest, 10 February 2016 [22]	Jack in the Box removes soda from its kids' menus.	Jack and the Box joined nine other major restaurants to remove SSBs as the default beverage from their children's meals and menus.
U.S. Food and Drug Administration, 6 May 2016 [23]	Guidance for Industry: A Labeling Guide for Restaurants and Retail Establishments Selling Away-From-Home Foods - Part II	This labeling guide is intended to help restaurants and similar retail food establishments understand nutrition labeling requirements under the U.S. Federal Food, Drug, and Cosmetic Act (FD&C Act) and implementing regulations that may apply to them.<br>The requirements include: the menu labeling provisions of section 403(q)(5)(H) of the (FD&C Act) [21 U.S.C. 343(q)(5)(H)] and the final rule on nutrition labeling of standard menu items in restaurants and similar retail food establishments we published on December 1, 2014 (79 FR 71156) (menu labeling final rule) that will take effect in May 2017.

Neuman, New York Times, 26 May 2016 [24]	New York City can enforce salt warnings on menus, court says.	A court cleared the way for NYC to enforce a rule requiring restaurants to inform consumers about food items sold with a high salt content. Some restaurant chains including Applebee's, TGI Friday's and Subway have voluntarily included a black and white salt icon that shows items containing greater than the recommended 2300 milligrams of sodium or a teaspoon of salt consumed per person per day. Other chains have not participated and the rule has been opposed by the NRA. The labeling rule applies to restaurants with at least 15 businesses nationwide.
Food and Drug Administration, 1 June 2016 [25]	FDA issues draft guidance to food industry for voluntarily reducing sodium in processed and commercially prepared food.	FDA issued draft guidance for public comment that provides practical, voluntary sodium-reduction targets for the food industry. The average sodium intake in the United States is 3,400 milligrams/day. The FDA offers draft short-term (two-year) and long-term (10-year) voluntary targets for industry to help the American public reduce sodium intake to 2,300 milligrams/day, which is recommended by experts based on scientific evidence.
Kick the Can, 8 June 2016 [26]	City of Stockton CA passes kids' beverage ordinance.	The city of Stockton passed an ordinance requiring either water or milk to be served as the default beverage in children's meals, the second law of its kind adopted by an American city, following the city of Davis, California. The so-called "healthy-by-default" rule – passed unanimously by the Stockton City Council – aims to tackle

		the city's skyrocketing childhood obesity rate and worsening type 2 diabetes epidemic.
Aguayo, Environmental Working Group, 12 August 2016 [27]	Panera Bread debuts 'Kids Meal Promise.'	In August 2016, Panera announced a pledge to offer children's meals that are free of artificial flavor, preservatives, sweeteners and colors and challenged the fast food and fast casual restaurant industry to promote children's health. As part of this pledge, Panera committed to offering:<br>• Clean food with no artificial flavors, preservatives, sweeteners and colors<br>• No toys or cartoon characters<br>• Wholesome, tasty choices<br>• Healthy side dishes including organic yogurt, sprouted grain rolls, carrots and apples instead of fries<br>• Water, organic milk and juice as the default beverage instead of soft drinks

Abbreviations

Food and Drug Administration (FDA), National Restaurant Association (NRA), New York City (NYC), Sugar-sweetened beverages (SSBs)

References

1. NRA joins Healthy Dining to promote healthful menu choices. *Fast Casual*. 17 July 2006. Available online: http://www.fastcasual.com/news/nra-joins-healthy-dining-to-promote-healthful-menu-choices/ (accessed 15 May 2017).
2. National Restaurant Association. *Produce and foodservice leaders set goal to double produce use in 10 years* 27 July 2009 [press release]. Available online: http://www.restaurant.org/Pressroom/Press-Releases/Produce-and-Foodservice-Leaders-Set-Goal-to-Double (accessed 15 May 2017).
3. National Restaurant Association. *National Restaurant Association says nutrition information provision is win for consumers and restaurants*. 22 March 2010 [news release]. Available online: (accessed 15 May 2017).
4. The White House Office of the First Lady. *Remarks by the First Lady in Address to the National Restaurant Association Meeting*. 13 September 2010 [press release]. Available online: http://www.whitehouse.gov/the-press-office/2010/09/13/remarks-first-lady-address-national-restaurant-association-meeting (accessed 15 May 2017).
5. Slossen M. Jack in the Box yanks toy from kids' meals. *Reuters*. 22 June 2011. Available online: http://www.reuters.com/article/us-toys-fastfood-idUSTRE75K6RZ20110622 (accessed 15 May 2017).
6. National Restaurant Association. *Kids, parents praise NRA's Kids LiveWell program*. 13 July 2011 [media release]. Available online: http://www.restaurant.org/News-Research/News/Kids,-parents-praise-NRA-s-Kids-LiveWell-program (accessed 15 May 2017).
7. Bryson York E. McDonald's to kids: Eat fruit, drink milk, visit Arches. New ads that include nutritional, physical activity message to debut Friday. *Chicago Tribune*. March 5, 2012. Available online: http://www.chicagotribune.com/business/ct-biz-0305-mcds-kids-ads-20120305,0,2269544.story (accessed 15 May 2017).
8. BlackPRWire (BPRW). *McDonald's USA's new happy meal campaign to engage families in the benefits of active play, balanced eating*. 6 March 2012. Available online: http://www.blackprwire.com/press-releases/3471-bprw_mcdonalds_usas_new_happy_meal_campaign_to_engage_families_in_the_benefits_of_active_play_balanced_eating (accessed 15 May 2017).
9. National Restaurant Association. *NRA honored for Kids LiveWell campaign*. 26 February 2013 [media release]. Available online: http://www.restaurant.org/News-Research/News/NRA-honored-for-Kids-LiveWell-campaign (accessed 15 May 2017).
10. Taco Bell. *Taco Bell becomes first quick service restaurant to discontinue kid's meals and toys nationally*. 23 July 2013 [news release]. Available online: http://www.tacobell.com/Company/newsreleases/Kids_Meals_Discontinued (accessed 15 May 2017).
11. Subway. *Subway Restaurants answers First Lady Michelle Obama's call to market healthier choices to kids*. 23 January 2014 [press release]. Available online: http://www.subway.com/PressReleases/PHA.pdf (accessed 15 May 2017).

12. Center for Science in the Public Interest. *Restaurant chains urged to dump soda from kids' menus*. January 30, 2014 [media release]. Available online: http://cspinet.org/new/201401301.html (accessed 15 May 2017).
13. O'Reilly L. McDonald's slapped down for focusing its Happy Meal advertising on the toy and not the food. *Business Insider*. May 15, 2015. Available online: http://www.businessinsider.com/mcdonalds-told-to-change-ads-by-childrens-advertising-review-unit-2015-5 (accessed 15 May 2017).
14. Center for Science in the Public Interest. *City of Davis, CA takes soda off kids' menus*. May 27, 2015 [news release]. Available online: http://cspinet.org/new/201505271.htm (accessed 15 May 2017).
15. McDonald's Corporation. *McDonald's and Alliance for a Healthier Generation Announce progress on commitment to promote balanced food and beverage choices*. June 25, 2015 [media release]. Available online: http://news.mcdonalds.com/press-releases/mcdonald-s-and-alliance-for-a-healthier-generation-announce-progress-on-commitme-nyse-mcd-1203234 (accessed 15 May 2017).
16. Food and Drug Administration. *FDA statement on extension of menu labeling compliance date*. 9 July 2015 [media release]. Available online: http://www.fda.gov/Food/NewsEvents/ConstituentUpdates/ucm453529.htm (accessed 15 May 2017).
17. Center for Science in the Public Interest. Applebee's to remove soda from kids menus. November 30, 2015 [media release]. Available online: http://www.cspinet.org/new/201511302.html (accessed 15 May 2017).
18. Aubrey A, Godoy M. High-sodium warnings hit New York City menus. *National Public Radio*. 1 December 2015. Available online: http://www.npr.org/sections/thesalt/2015/12/01/458031755/in-new-york-city-that-salty-combo-meal-now-comes-with-a-warning (accessed 15 May 2017).
19. National Restaurant Association. NRA sues NYC over sodium-labeling mandate. 4 December 2015 [press release]. Available online: http://www.restaurant.org/News-Research/News/NRA-sues-NYC-over-sodium-labeling-mandate (accessed 15 May 2017).
20. Madhani A. McDonald's nears sale of 2 billion servings of fruit, yogurt. *USA Today*. January 19, 2016. Available online: http://www.usatoday.com/story/money/2016/01/19/mcdonalds-nears-sale-2-billion-servings-fruit-yogurt/79018448/ (accessed 15 May 2017).
21. Gartland M. Lawmakers attempt at 'healthy' Happy Meals falls short. *New York Post*. January 27, 2016. Available online: http://nypost.com/2016/01/27/lawmakers-attempt-at-healthy-happy-meals-falls-short/ (accessed 15 May 2017).
22. Center for Science in the Public Interest. *Jack in the Box removes soda from its kids' menus*. February 10, 2016 [media release]. Available online: http://cspinet.org/new/201602101.html (accessed 15 May 2017).
23. Food and Drug Administration. *Guidance for Industry: A Labeling Guide for Restaurants and Retail Establishments Selling Away-From-Home Foods - Part II (Menu Labeling Requirements in Accordance with 21 CFR 101.11)*. U.S. Department of Health and Human Services, Food and Drug Administration, Center for Food Safety and Applied Nutrition. 6 May 2016. Available online: http://www.fda.gov/Food/GuidanceRegulation/GuidanceDocumentsRegulatoryInformation/ucm461934.htm (accessed 15 May 2017).

24. Newman W. New York City can enforce salt warnings on menus, court says. *The New York Times*. 26 May 2016. Available online: http://www.nytimes.com/2016/05/27/nyregion/new-york-city-can-enforce-salt-warnings-on-menus-court-says.html?_r=0 (accessed 15 May 2017).
25. Food and Drug Administration. *FDA issues draft guidance to food industry for voluntarily reducing sodium in processed and commercially prepared food*. 1 June 2016 [news release]. Available online: http://www.fda.gov/NewsEvents/Newsroom/PressAnnouncements/ucm503874.htm (accessed 15 May 2017).
26. Kick the Can. *City of Stockton CA passes kids' beverage ordinance*. June 8, 2016 [media release]. Available online: http://www.kickthecan.info/city-stockton-ca-passes-kids%E2%80%99-beverage-ordinance (accessed 15 May 2017).
27. Aguayo J. *Panera Bread debuts 'Kids Meal Promise.'* Environmental Working Group blog. 12 August 2016. Available online: https://www.fastcasual.com/news/panera-bread-debuts-kids-meal-promise/ (accessed 15 May 2017).
